# Attenuated Structural Transformation of Aconitine during Sand Frying Process and Antiarrhythmic Effect of Its Converted Products

**DOI:** 10.1155/2021/7243052

**Published:** 2021-10-25

**Authors:** Yu-Jie Wang, Pei Tao, Yan Wang

**Affiliations:** ^1^School of Ethnic Medicine, Chengdu University of Traditional Chinese Medicine, Chengdu 611137, China; ^2^School of Pharmacy, Chengdu University of Traditional Chinese Medicine, Chengdu 611137, China

## Abstract

The transformation pathways of diterpenoid alkaloids have been clarified in the boiling and steaming process. Aconitine, a famous diterpenoid alkaloid, is successively transformed into benzoylaconine and aconine during the processes of boiling and steaming, but the transformation pathway remains to be determined in the sand frying process. The present study aims at investigating the transformation pathways of aconitine in the process of sand frying, as well as assessing the cardiotoxicity and antiarrhythmic activity of aconitine and its converted products. The parameters of temperature and time for the structural transformation of aconitine were confirmed by HPLC. The converted products were further separated and identified by column chromatography, NMR, and HR-ESI-MS. Furthermore, by observing the lead II electrocardiogram (ECG) changes in rats under an equivalent dose, the cardiotoxicity of aconitine and its converted products were compared. Ultimately, the antiarrhythmic effect of the converted products was investigated by employing the model of aconitine-induced arrhythmia. Consequently, the structure of aconitine was converted when processed at 120°C–200°C for 1–40 min. Two diterpenoid alkaloids, a pair of epimers, namely, pyroaconitine and 16-epi-pyroaconitine, were further isolated from processed aconitine. 0.03 mg/kg aconitine induced arrhythmias in normal rats, while the converted products did not exhibit arrhythmias under an equal dose. In the antiarrhythmic assay, 16-epi-pyroaconitine could dose-dependently delay the onset time of VPB, reduce the incidence of VT, and increase the arrhythmia inhibition rate, demonstrating comparatively strong antiarrhythmic activity. Conclusively, compared with the prototype compound aconitine, the converted products exhibited lower cardiotoxicity. Further investigations on the cardiotoxicity indicated that pyroaconitine with *β* configuration had a stronger cardiotoxicity than 16-epi-pyroaconitine with *α* configuration. Furthermore, 16-epi-pyroaconitine could antagonize the arrhythmogenic effect caused by the prototype compound aconitine; the antiarrhythmic effect of 16-epi-pyroaconitine was stronger than lidocaine and propafenone, which had the potential to be developed as antiarrhythmic drugs.

## 1. Introduction

There are more than 300 species of *Aconitum* plants belonging to the family of Ranunculaceae worldwide, mainly distributed in the temperate regions of the northern hemisphere, of which over 200 species are distributed in China, most of them are found in Sichuan, Yunnan, and Tibet Autonomous Region [1–4]. 76 species are diffusely applied in traditional Chinese medicine and ethnomedicine [2], such as *Aconitum pendulum* Busch, *A. carmichaelii* Debx., *A. kusnezoffii* Reichb., and *A. coreanum* (H. Lév.) Rapaics [5, 6]. They are widely used in prescriptions for treating ankle pains, rheumatoid arthritis, and traumatic injuries [6, 7]. The key obstacle for their medical application may be attributed to the extremely high toxicity.

Diterpenoid alkaloids, including aconitine, hypaconitine, and 3-acetylaconitine, are the dominant active constituents of unprocessed *Aconitum* plants [8, 9]. These ingredients are highly toxic, which induce arrhythmia, respiratory spasm, numbness of limbs, asphyxia, and are even life-threatening [8]. Therefore, processing is necessary for poisonous *Aconitum* drugs. Currently, two main methods are applied for the processing of *Aconitum* herbs in traditional Chinese medicine, namely, boiling with water for 4–6 hours and steaming for 6–8 hours. The processing mechanism of the above methods is that diester diterpenoid alkaloids are sequentially hydrolyzed into less toxic monoester- and alkanolamine-diterpenoid alkaloids. For example, aconitine is successively transformed into benzoylaconine and aconine during the processes of boiling and steaming [3, 10]. Different from boiling and steaming, the method of sand frying is a unique processing method for *Aconitum* herbs in ethnomedicine; it only takes 10–15 minutes of stir-frying with sand to achieve the purpose of detoxification. However, the processing mechanism during processing is quite different. The structural transformation pathways of diester diterpenoid alkaloids are more complicated during the sand frying process, and the transformation laws have not been fully elucidated [11–13]. To clarify the specific structural transformation pathways of diterpenoid alkaloids, a classical and well-known diterpenoid alkaloid aconitine was chosen as the research object.

Furthermore, it is worth noting that some structurally similar diterpenoid alkaloids exert opposite effects. For example, aconitine has fatal arrhythmogenic effects, while its structurally similar alkaloids 14-benzoyltalatisamine, 14-benzoyldelcosine, and 14-benzoylidocarpine [14–16] have antiarrhythmic effects. It is hypothesized that, with the transformation of structure, the transformed components may generate antiarrhythmic effects. Besides, aconitine-induced arrhythmia is a classical model for testing the efficacy of antiarrhythmic agents. Therefore, we ultimately investigated the antiarrhythmic activity of the transformed components by employing a rat model of aconitine-induced arrhythmia.

## 2. Materials and Methods

### 2.1. General Experimental Procedures

The lead II electrocardiograms (ECGs) were recorded using BL-420F Organism Functional Experimental System (Chengdu Techman Software Co., Ltd., Chengdu, China). CPA2250 electronic analytical balance (Sartorius, Germany) was used to correctly weigh the lab samples. The process of sand frying was simulated by oil bath heating using an HH-SJ heat-collecting magnetic stirrer (Jintan Chengdong Xinrui Instrument Factory, Changzhou, China). Optical rotations were obtained on a Perkin-Elmer 341 polarimeter (Perkin-Elmer, USA). Melting point was measured on an X-4 micro-melting point apparatus and uncorrected (Shanghai Precision Scientific Instrument Corporation, Shanghai, China). NMR spectra were measured on a Bruker Avance 600 spectrometer (Bruker, Germany) in CDCl_3_ with tetramethylsilane (TMS) as the internal standard. Mass spectra were carried out on a micrOTOF-Q-II mass spectrometer (Bruker, Germany). Silica gel G (200–300 mesh) for column chromatography and TLC plates (silica gel G) were obtained from Qingdao Sea Chemical Factory (Qingdao, China). Spots on TLC plates were visualized with Dragendorff's reagent.

### 2.2. Chemicals and Reagents

Aconitine (purity = 99%) was purchased from Shaanxi Herbchem Biotech Co., Ltd. (Xi'an, China). Propafenone hydrochloride (purity = 99.8%) and lidocaine hydrochloride (purity = 93.4%) were purchased from National Institutes for Food and Drug Control (Beijing, China), urethane from Chengdu Kelong Chemical Reagent Factory (Chengdu, China). Pyroaconitine (purity = 97.7%) and 16-epi-pyroaconitine (purity = 98.1%) were isolated and identified from the sand-fried processed product of aconitine; the structures were verified by ^1^H-NMR, ^13^C NMR, and HR-ESI-MS.

HPLC-grade acetonitrile (purity = 99.9%) was supplied by Fisher Chemical Co. (New Jersey, USA). Ultrapure water was directly obtained from a ULUP-I-10T water purification system (Chengdu, China). The remaining reagents were all of analytical grade.

### 2.3. Animals

SPF-grade Sprague Dawley (SD) rats with either sex weighing between 180 g and 220 g (certificate no. SCXK (CHUAN) 2015–030) were supplied from Chengdu Dossy Experimental Animals Co., Ltd. (Chengdu, China) and maintained under a controlled light/dark cycle and temperature (20 ± 2°C), with free access to food and water. They were left for 2 d for acclimatization to animal room conditions. Animal experiments were performed in adherence with the Guiding Principles for the Care and Use of Laboratory Animals of China and were approved by the Animal Experimentation Ethics Committee of Chengdu University of Traditional Chinese Medicine (permission no. 2020–15).

### 2.4. Screening of Processing Parameters

#### 2.4.1. Chromatographic Conditions

Quantitative analyses were conducted with an Agilent 1260 system, using a COSMOSIL PAK 5C_18_-MS-II column (250 × 4.6 mm, 5 *μ*m) maintained at 30°C. The mobile phase composition was (A): acetonitrile and (B): 0.03 moL/L NH_4_HCO_3_ (the aqueous was adjusted to pH 9.5 using 25% NH_4_OH) (A: 39%, B: 61%, v/v) used at a flow rate of 1 mL/min. The injection volume was 10 *μ*L and the detection wavelength was 230 nm.

#### 2.4.2. Preparation of Standard Solution

The stock solution was prepared by dissolving 100 mg aconitine in dichloromethane to a final concentration of 0.40 mg/mL. 4 mL of the stock solution was added into a 10 mL volumetric flask, the solvent was subsequently evaporated, and 0.1% HCl-methanol was added for dissolution to obtain a standard solution with 160 *μ*g/mL.

#### 2.4.3. Preparation of the Processed Product Sample Solution

Fifty 100 mL round-bottomed flasks were divided into five temperature groups (120°C, 140°C, 160°C, 180°C, and 200°C), and ten reaction time points (1, 3, 5, 10, 15, 20, 25, 30, 35, and 40 min) were set at each temperature. Adding 4 mL of the stock solution into each flask, evaporate the solvent under reduced pressure. The flasks were subsequently immersed in an oil bath and processed according to set parameters, cooling to room temperature after the reaction. The residue was diluted with 0.1% HCl-methanol in a 10 mL volumetric flask, subsequently filtered with 0.45 *μ*m syringe filter before injecting into the HPLC.

#### 2.4.4. Method Validation

*(1) Linearity, Limits of Detection, and Quantification*. The stock solution for linearity was prepared by dissolving an accurately weighed amount of aconitine in 0.1% HCl-methanol, achieving a concentration of 2.0 mg/mL. Accurately adding 0.025, 0.1, 0.2, 0.4, 0.8, 1.0, and 2.0 mL of stock solution into per 10 mL flask, 0.1% HCl-methanol was added for dissolution to prepare solutions with final concentrations of 5, 20, 40, 80, 160, 200, and 400 *μ*g/mL. The calibration curve was constructed by plotting the peak areas (*Y*) against the corresponding concentrations (*X*). Limit of detection (LOD) and limit of quantification (LOQ) were confirmed by injecting a series of standard solutions till the signal-to-noise ratio (S/N) was 3 and 10 for LOD and LOQ, respectively.

*(2) Precision*. Precision of the method was determined by multiple injection (6 times) of the same standard solution.

*(3) Stability*. The stability was tested by replicate injection of the same standard solution after stored at 4°C for 0, 2, 4, 8, 12, 24, 36, 48, 60, and 72 h, respectively.

*(4) Repeatability*. The repeatability was evaluated by injecting six independent samples. The heating parameters were set at 140°C for 10 min.

### 2.5. Preparation and Separation of the Converted Products

600 mg aconitine was dissolved in a 250 mL round-bottomed flask with an appropriate amount of dichloromethane. To make the sample uniformly adhere to the inner wall of the flask, the solvent was removed under reduced pressure to no dichloromethane. The flask was subsequently immersed in an oil bath and heated at 160°C for 25 min, cooling to room temperature after reaction, obtaining the processed product of aconitine (560 mg) for column chromatography.

The residue was subjected to column chromatography (silica gel, 120 g, 200–300 mesh) and eluted with petroleum ether-acetone-triethylamine 6 : 1:0.01 (0.7 L), 3 : 1:0.01 (2.4 L) to obtain three fractions (A–C). Fraction B (190 mg) and fraction C (260 mg) were further purified on silica gel column (120 g, 200–300 mesh), eluting with petroleum ether-acetone-triethylamine 3 : 1:0.01 to afford compounds **1** (100 mg) and **2** (115 mg), respectively.

### 2.6. Electrocardiography

The animals were anesthetized by intraperitoneal (i.p.) injection of 20% urethane (1.2 g/kg), with their back fixed and four limbs in subcutaneous penetration of needle electrodes. The surface lead II ECGs were begun to document after the administration of urethane.

### 2.7. Cardiotoxicity Test

**Figure 1:**
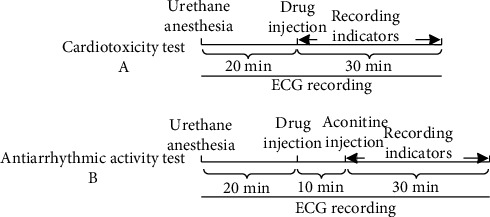
Experimental timeline.

Thirty rats were randomly divided into three groups: aconitine, pyroaconitine (compound **1**), and 16-epi-pyroaconitine (compound **2**). It was found that 0.03 mg/kg aconitine caused ventricular premature beat (VPB), ventricular tachycardia (VT), and ventricular fibrillation (VF) in normal rats. Whether equivalent converted products caused arrhythmias can directly reflect the structural changes of the prototype compound on its cardiotoxicity [11] (Figure 1).

### 2.8. Aconitine-Induced Arrhythmia Test

141 SD rats were randomly divided into 8 groups, the grouping is shown in Table 1. The blank solvent was prepared by taking 4 mL of 1% HCl-ethanol and adding saline for volume fixation to 100 mL. The preparation of aconitine, positive drugs, and 16-epi-pyroaconitine was the same as the blank solvent. To ascertain whether the blank solvent would influence the ECGs of rats, the rats of the solvent group were only administered the same volume of blank solvent to observe the ECG changes during the recording time.

The rats were anesthetized using 20% urethane (1.2 g/kg, i.p.) [19]. Recording the lead II ECGs for 20 min before drug administration; then, 16-epi-pyroaconitine, positive drugs, and equal volume of saline were subsequently administered via vena femoral, respectively. After stabilization for 10 min [20], 0.03 mg/kg aconitine [21–23] was injected into the vena femoral to trigger arrhythmia (Figure 1). The onset time of VPB [24] was recorded within 30 min [24] after aconitine administration. The VT [25] or arrhythmia [26], if any, was also recorded for each group at the end of the observation period.

### 2.9. Statistical Analysis

The data obtained were analyzed using the Statistical Package for Social Sciences (SPSS) version 20 software. Experimental data were expressed as mean ± SD or proportion. Descriptive statistics were examined individually. When the data conformed to the normal distribution, one-way ANOVA would be used for those with homogeneous variance, if the variance was not homogeneous. Tamhane's T2 test would be used for comparison between groups. Comparisons of proportions were made with the Pearson chi-square (*χ*^2^) test. *P* < 0.05 was considered to be a statistically significant difference.

## 3. Results and Discussion

### 3.1. Method Validation

The obtained results of standard sample showed that aconitine exhibited good linearity within test ranges (*Y* = 11461*X*-39.79, *r* = 0.9995). LOD and LOQ for aconitine under the present chromatographic condition were 0.64 *μ*g/mL and 2.06 *μ*g/mL, respectively.

The RSD values of aconitine for the precision, stability, and repeatability were 0.94%, 0.86%, and 1.65%, respectively, indicating the established method was precise, accurate, and sensitive enough for determination.

### 3.2. Temperature and Time for the Structural Transformation of Aconitine

**Figure 2:**
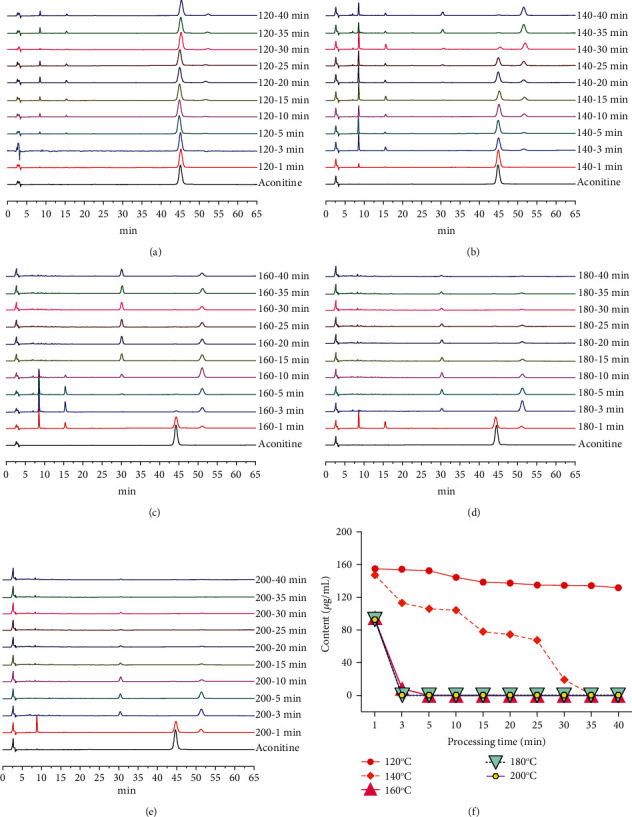
Chromatograms and contents of aconitine under different processing temperatures and time. (a–e) HPLC chromatograms of different processed products of aconitine. (a) 120°C. (b) 140°C. (c) 160°C. (d) 180°C. (e) 200°C. (f) Content variation of aconitine under different processing temperatures and time.

To screen out the temperature and time range for the structural transformation of aconitine during processing, the sample solutions of different processing temperatures and time, together with the standard solutions, were injected into the HPLC, respectively. The chromatograms were obtained after determination, which could visually reflect the dynamic variation of aconitine, as vividly shown in Figure 2.

The content of aconitine was 160.00 *μ*g/mL before processing, decreased to 19.36 *μ*g/mL after processing at 140°C for 30 min, and dropped to 0 *μ*g/mL after continuous heating for 5 min. When processed at 160°C for 3 min, the content steeply declined to 8.26 *μ*g/mL, even dropped to 0 *μ*g/mL for another 2 min (Table 2). Furthermore, four unknown chromatographic peaks emerged in the chromatograms of 120°C (15–40 min), 140°C (3–40 min), 160°C (1–40 min), 180°C (1–35 min), and 200°C (1–20 min) with retention times of 8 min, 15 min, 30 min, and 51 min, indicating that there were at least four transformed products of aconitine during processing.

### 3.3. Structural Identification of the Converted Products

Compounds **1** and **2** were isolated from processed product of aconitine by procedures as described in the experimental section.

Pyroaconitine (**1**) was obtained as a white amorphous powder, [*α*]_D _^20^+ 30.8° (*c* = 1.02, CH_3_OH). Its molecular formula was deduced to be C_32_H_43_NO_9_ from a pseudomolecular ion at m/*z* 586.3010 [M + H]^+^ (calcd. 586.3011) in its HR-ESI-MS. The NMR spectra (Table 3) showed the presence of an *N*-ethyl group (*δ*_H_ 1.01, 3H, *t*, *J* = 7.32 Hz; *δ*_H_ 2.41, 2.44, each 1H, *m*; *δ*_C_ 13.3 *q*, 49.1 t), four methoxyl groups (*δ*_H_ 3.23, 3.27, 3.61, 3.28, each 3H, *s*; *δ*_C_ 56.1 *q*, 57.9 *q*, 61.7 *q*, 59.2 q), a benzoyl group (*δ*_H_ 7.40, 2H, *t*, *J* = 7.32 Hz; 7.53, 1H, *t*, *J* = 7.32 Hz; 7.94, 2H, *d*, *J* = 7.32 Hz; *δ*_C_ 167.1 s, 129.6 s, 129.8 d (2C), 128.4 d (2C), 133.3 d), as well as a carbonyl group (*δ*_C_ 211.8 s). These data suggested that compound **1** was a pyro-type alkaloid [27].

**Figure 3:**
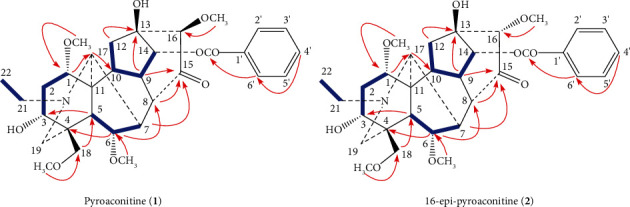
Key ^1^H–^1^H COSY correlations (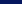) and HMBC correlations (H
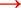
C) of compounds **1** and **2**.

The ^1^H-doublet signal at *δ*_H_ 5.16 (*J* = 5.1 Hz) was assigned to H-14*β*, resulting in location of the benzoyl group at C-14 [28, 29]. Four methoxyl groups were attributed to C-1, C-6, C-16, and C-18 based on the cross-peaks between 1-OCH_3_ (*δ*_H_ 3.23, s) and C-1 (*δ*_C_ 83.5, d), 6-OCH_3_ (*δ*_H_ 3.27, s) and C-6 (*δ*_C_ 84.1, d), 16-OCH_3_ (*δ*_H_ 3.61, s) and C-16 (*δ*_C_ 89.2, d), 18-OCH_3_ (*δ*_H_ 3.28, s) and C-18 (*δ*_C_ 76.2, t) in its HMBC spectrum (Figure 3). Two hydroxyl groups were assigned to C-3 and C-13 based on the correlations between the C-3 (*δ*_C_ 71.5, d) and H-5 (*δ*_H_ 2.03), H-18 (*δ*_H_ 3.68, 3.75), H-19 (*δ*_H_ 2.37, 2.89), as well as C-13 (*δ*_C_ 76.5, s) and H-9 (*δ*_H_ 2.78), H-12 (*δ*_H_ 2.26, 2.71), H-14 (*δ*_H_ 5.16) in the HMBC of **1**.

**Figure 4:**
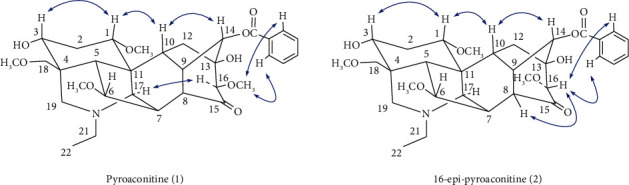
Key NOE correlations (H
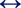
H) of compounds **1** and **2**.

The key NOE correlations (Figure 4) could be observed between H-16 and H-17, 16-OCH_3_ and H-2′, 6′ in compound **1**. As a result, the configuration of the 16-OCH_3_ in compound **1** was unambiguously established to have a *β*-orientation. Comparison of the NMR spectra of **1** with those of aconitine [30], it could be found that compound **1** was lack of an acetoxyl group. The ^13^C NMR spectra of **1** and aconitine were very similar except for the chemical shifts of C-8 signal appeared at a higher field, caused by the absence of an acetoxyl group. Furthermore, the carbonyl group was assigned to C-15 based on the long-range correlations between C-15 (*δ*_C_ 211.8, s) and H-8 (*δ*_H_ 2.75), H-9 (*δ*_H_ 2.78), and H-16 (*δ*_H_ 3.29) in the HMBC spectrum of **1** (Figure 3). All of the above arguments determined the structure of **1** as pyroaconitine (Figures S1–S12).

16-Epi-pyroaconitine (**2**) was obtained as colorless needles, mp 164–166°C, [*α*]_D_^20^ − 101.1 (*c* = 1.00, CH_3_OH). The pseudomolecular ion at *m/z* 586.3008 [M + H]^+^ (cacld. 586.3011) in its HR-ESI-MS spectrum suggested a molecular formula of C_32_H_43_NO_9_. ^1^H and ^13^C NMR spectra of **2** (Table 4) showed the distinct NMR features of an aconitine-type alkaloid skeleton [31], bearing an *N*-ethyl group (*δ*_H_ 1.04, 3H, *t*, *J* = 7.38 Hz; *δ*_H_ 2.45, 2.51, each 1H, *m*; *δ*_C_ 13.3 *q*, 49.0 t), four methoxyl groups (*δ*_H_ 3.24, 3.24, 3.80, 3.29, each 3H, *s*; *δ*_C_ 56.1 *q*, 57.8 *q*, 62.3 *q*, 59.2 q), a benzoyl group (*δ*_H_ 7.46, 2H, *t*, *J* = 7.32 Hz; 7.60, 1H, *t*, *J* = 7.32 Hz; 7.97, 2H, *d*, *J* = 7.32 Hz; *δ*_C_ 166.1 s, 129.4 s, 129.7 d (2C), 128.6 d (2C), 133.6 d), as well as a carbonyl group (*δ*_C_ 211.7 s).

The one-proton double signal (*J* *=* 5.16 Hz) at *δ*_H_ 5.41 was attributed to H-14*β*, implying the appearance of a benzoyl group at C-14 position [28, 29]. The location of four methoxyl groups were based on the long-range correlations between 1-OCH_3_ (*δ*_H_ 3.24) and C-1 (*δ*_C_ 83.6 d), 6-OCH_3_ (*δ*_H_ 3.24) and C-6 (*δ*_C_ 84.1 d), 16-OCH_3_ (*δ*_H_ 3.80) and C-16 (*δ*_C_ 86.1 d),18-OCH_3_ (*δ*_H_ 3.29) and C-18 (*δ*_C_ 76.8 t) in HMBC spectrum (Figure 3). Furthermore, two hydroxyl groups were assigned to C-3 and C-13 based on the correlations between the C-3 (*δ*_C_ 71.8, d) and H-5 (*δ*_H_ 2.04), H-18 (*δ*_H_ 3.69, 3.75), H-19 (*δ*_H_ 2.39, 2.90), as well as C-13 (*δ*_C_ 77.5, s) and H-9 (*δ*_H_ 2.80), H-12 (*δ*_H_ 1.93, 3.01), H-14 (*δ*_H_ 5.41) in the HMBC of **2**.

The ^1^H- and ^13^C NMR spectra of **2** were almost the same as those of **1**, with the exception of the chemical shift assignable to the methoxyl group at C-16; *δ* 3.85 and 3.29 in the ^1^H-NMR spectra of **2** and **1**, respectively. The ^1^H- and ^13^C NMR signals at C-16 of **2** were shifted 0.56 ppm downfield and 3.1 ppm upfield in comparison with those of **1**, respectively. On the basis of the ^1^H-NMR spectrum and consideration of the stereochemistry in the molecular model, the methoxy protons at C-16 in **1** were affected by the shielding effect of the aromatic ring, but the C-16 methoxy group in **2** was not [27, 31, 32]. These data suggested that compound **2** was an epimer to compound **1** at C-16 position. The NOE correlations (Figure 4) between H-16 and H-2′, 6′, H-8 also confirmed the configuration of 16-OCH_3_ in **2** has an *α*-orientation. Thus, the structure of **2** was determined to be 16-epi-pyroaconitine (Figures S13–S28).

### 3.4. Cardiotoxicity of Aconitine and Its Converted Products

**Figure 5:**
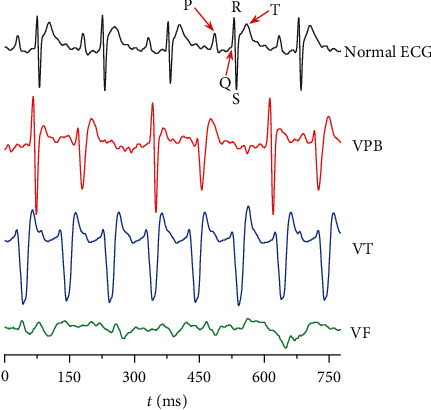
Representative ECGs of SD rats.

0.03 mg/kg aconitine-induced arrhythmias in normal rats, such as VPB, VT, and VF, were accompanied by regular chest twitching. In contrast, no obvious abnormal ECG features were recorded in groups of pyroaconitine and 16-epi-pyroaconitine under the same dose. These results demonstrated that the cardiotoxicity of the converted products was reduced compared with aconitine, achieving the purpose of reducing toxicity (Table 5 and Figure 5). It is noteworthy that the dose of pyroaconitine increased to 0.13 mg/kg could induce VPB and VT in normal rats, even 10% of the rats died of fatal arrhythmias, exhibiting an arrhythmogenic effect. Comparatively, the cardiotoxicity of pyroaconitine was stronger than 16-epi-pyroaconitine.

### 3.5. Antiarrhythmic Effects of the Converted Product

#### 3.5.1. Effect of 16-Epi-Pyroaconitine on VPB Incubation Period

VPB is the initial manifestation of aconitine-induced arrhythmia model rats, characterized by premature and bizarrely shaped QRS complexes that appear wide on the ECG, without *P* wave, and a *T* wave is usually oriented in a direction opposite the major deflection of the QRS [33]. VPB incubation period refers to the time after the injection of aconitine to the first occurrence of VPB [24]; the longer the incubation period is, the better the antiarrhythmic effect is.

In the control group, the typical characteristic ECGs of VPB and VT appeared after aconitine injection; even part of the rats developed to VF and lasted for more than 30 min, indicating that the arrhythmia model was established successfully. The VPB incubation period of aconitine-induced arrhythmia was (97.7 ± 23.7) *s*.

Lidocaine and propafenone delayed the onset time of VPB induced by aconitine, and the incubations of VPB were (180.9 ± 51.0) *s* and (142.3 ± 17.5) *s*, respectively. Compared with the control group, two positive drugs had a significant difference (*P* < 0.05).

**Figure 6:**
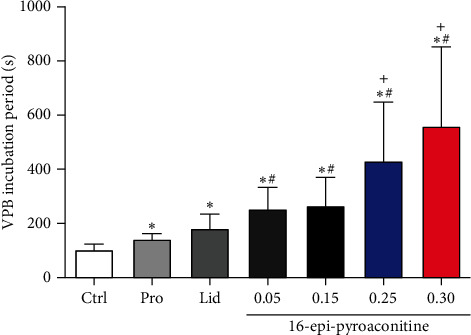
Effect of 16-epi-pyroaconitine on VPB incubation period. Data are presented as mean ± SD. ^*∗*^*P* < 0.05, compared with control group; ^+^*P* < 0.05, compared with the lidocaine group; ^#^*P* < 0.05, compared with the propafenone group. Ctrl: the control group. Lid: lidocaine. Pro: propafenone.

The effect of 16-epi-pyroaconitine on the latency of VPB was shown in Figure 6. The VPB incubation periods for different dose groups of 16-epi-pyroaconitine (0.05 mg/kg, 0.15 mg/kg, 0.25 mg/kg, and 0.30 mg/kg) were (252.7 ± 79.9) *s*, (263.9 ± 103.5) *s*, (430.1 ± 217.8) *s*, and (560.4 ± 288.5) *s*, respectively. Compared with the control group and propafenone, the onset time of VPB in all dose groups had a significant difference (*P* < 0.05). Moreover, compared with the positive drug lidocaine, there was an obvious difference in the 0.25 mg/kg and 0.30 mg/kg groups (*P* < 0.05).

#### 3.5.2. Effect of 16-Epi-Pyroaconitine on the Incidence of VT

VT is the result of further development of VPB, as well as an important factor in sudden cardiac death. The incidence of VT can be used to evaluate whether the experimental compound can effectively prevent the progress of VPB; the lower the occurrence is, the better the efficacy is.

**Figure 7:**
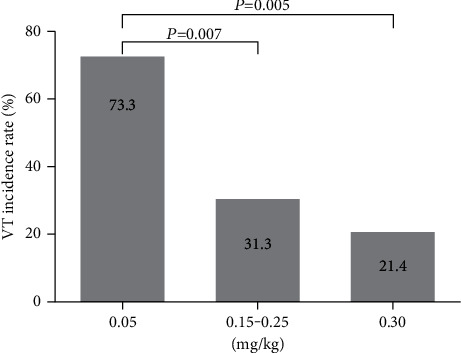
Effect of 16-epi-pyroaconitine on the incidence of VT.

Complying with the data analysis requirements of chi-square test, the adjacent dose groups with similar incidence should be combined to analyze. For this reason, four dose groups of 16-epi-pyroaconitine were partially combined and subsequently divided into three subgroups, namely, 0.05 mg/kg, 0.15–0.25 mg/kg, and 0.30 mg/kg dose groups. The chi-square test showed that the incidence of VT in the aforementioned three groups was significantly different (*χ*^2^ = 10.023, *P*=0.007), as illustrated in Table 6 and Figure 7. Compared with the 0.05 mg/kg group (73.3%), the occurrence of VT in groups of 0.15–0.25 mg/kg and 0.30 mg/kg was significantly declined to merely 31.3% and 21.4%, respectively (*χ*^2^ = 7.318, *P*=0.007; *χ*^2^ = 7.813, *P*=0.005).

#### 3.5.3. Effect of 16-Epi-Pyroaconitine on Arrhythmia Inhibition Rate

The definition for the incidence of arrhythmia [26] is the proportion of arrhythmia that occurs within 30 min after preinjection of test compounds, and an arrhythmia model was established with aconitine. Any kinds of abnormal ECGs, such as VPB, VT, or VF should be considered as arrhythmias. Arrhythmia inhibition rate (%) = 100% − arrhythmia incidence rate (%), that is, the proportion that no arrhythmia occurs within 30 min, which can be used to evaluate whether the experimental compound could antagonize the arrhythmogenic effect of aconitine. Arrhythmia inhibition rate is the most intuitive index reflecting the strength of the drug efficacy; the higher it gets, the better the efficacy is.

**Figure 8:**
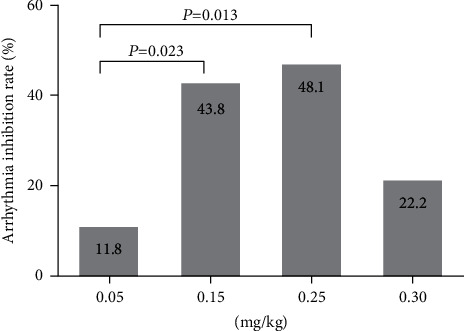
Effect of 16-epi-pyroaconitine on arrhythmia inhibition rate.

The effect of different dose groups of 16-epi-pyroaconitine on the arrhythmia inhibition rate in rats was analyzed. The results of chi-square test showed that there was a significant difference in the arrhythmia inhibition rate among the four groups (*χ*^2^ *=* 8.442, *P*=0.038; Table 7). Compared with an 11.8% arrhythmia inhibition rate in 0.05 mg/kg group, the arrhythmia inhibition rates were significantly increased to 43.8% and 48.1% in 0.15 mg/kg and 0.25 mg/kg groups, respectively (*χ*^2^ *=* 5.165, *P*=0.023; *χ*^2^ *=* 6.146, *P*=0.013), indicating that 16-epi-pyroaconitine could dose-dependently increase the arrhythmia inhibition rate, as depicted in Figure 8.

## 4. Discussion

Aconitine, the main ingredient of *A. pendulum* Busch and other *Aconitum* herbs, was chosen as the research object. The experiment results showed that aconitine was converted into four components when processed at 120°C–200°C for 1–40 min. Among them, two converted products, a pair of epimers, pyroaconitine and 16-epi-pyroaconitine, were further isolated and identified. Besides, 16-epi-pyroaconitine has been isolated from the sand-fried processed *A. pendulum* Busch in our previous study [13], these results demonstrated that the oil bath heating method could simulate and simplify the process of sand frying, truly reflecting the dynamic change process of the experimental compounds.

**Figure 9:**
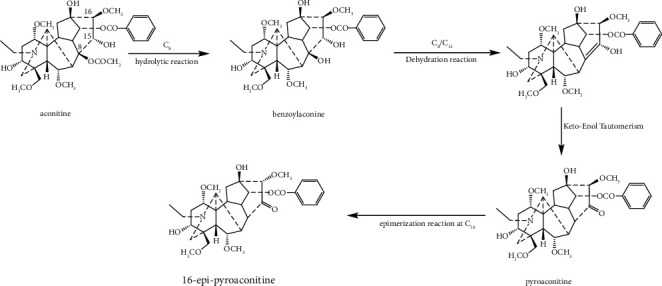
The structural transformation pathway of aconitine during the process of sand frying.

Comparing the structures of the prototype compound and its converted products, it can be found that the transformation pathways of aconitine during the sand frying process are different from those of the boiling and steaming methods. Firstly, the acetoxyl group at C-8 of aconitine was hydrolyzed to a hydroxyl group to obtain benzoylaconine. Then, the hydroxyl group at C-8 and the hydrogen atom at C-15 of benzoylaconine were further dehydrated, forming an enol structure, which was unstable and easily converted to its tautomeric isomer pyroaconitine. Finally, parts of pyroaconitine were transformed into 16-epi-pyroaconitine by the epimerization at C-16 (Figure 9).

By comparing the structures of pyroaconitine and 16-epi-pyroaconitine, it was found that they differed only in the –OCH_3_ configuration at the C-16 position. To clarify the effect of the 16-OCH_3_ configuration on the cardiotoxicity and antiarrhythmic effect, therefore, some relevant experiments on these two transformed compounds were further conducted.

The cardiotoxicity test showed that 0.03 mg/kg aconitine induced arrhythmias in normal rats. Meanwhile, the two converted products, pyroaconitine and 16-epi-pyroaconitine, did not exhibit arrhythmias during the observation period, demonstrating that the sand frying method could attenuate the cardiotoxicity of aconitine. In the antiarrhythmic activity assay, when preinjected 0.15 mg/kg pyroaconitine, the VPB incubation period was found to be shorter than the control group, suggesting that pyroaconitine could aggravate the arrhythmogenic effect of aconitine, which led to the advancement of arrhythmia onset. Therefore, further studies on the cardiotoxicity of pyroaconitine were conducted. The results indicated that pyroaconitine induced arrhythmias at 0.13 mg/kg, with a latency of VPB being (134.1 ± 36.5) *s*, implying that the cardiotoxicity may be stronger when the 16-position substituent was in *β* configuration than in *α* configuration.

The antiarrhythmic results demonstrated that 16-epi-pyroaconitine could dose-dependently delay the onset time of VPB, decline the occurrence of VT, and increase the arrhythmia inhibition rate. For example, the VPB incubation periods at doses of 0.25 mg/kg and 0.30 mg/kg were significantly longer than the positive drugs propafenone and lidocaine (*P* < 0.05, Figure 6). Similarly, it was also demonstrated that the incidence of VT has significantly dropped from 73.3% in 0.05 mg/kg to only 21.4% in 0.30 mg/kg (Figure 7). Moreover, at the dose of 0.15 mg/kg and 0.25 mg/kg, the arrhythmia inhibition rate was significantly increased to 43.8% and 48.1%, respectively (Figure 8), indicating that 16-epi-pyroaconitine has a comparatively strong antiarrhythmic activity.

## 5. Conclusions

In summary, this paper used the oil bath heating method to simulate the sand frying process and screened out the temperature and time parameters for the structural transformation of aconitine. In addition, it also demonstrated from the *in vivo* experiments that, with the structural transformation of aconitine, the cardiotoxicity of the converted products was reduced. Comparatively, the cardiotoxicity of pyroaconitine was stronger than that of 16-epi-pyroaconitine, which exhibited relatively strong antiarrhythmic effects.

## Figures and Tables

**Table 1:** Grouping of the experimental animals.

No.	Group	Dosage (mg/kg)	Number (*n*)
1	Blank solvent	—	10
2	Control	Saline	10
3	Positive	Lidocaine; 5.0 [17]	18
4		Propafenone; 3.2 [18]	9
5	16-Epi-pyroaconitine	0.05	17
6		0.15	32
7		0.25	27
8		0.30	18

**Table 2:** Content of aconitine under different processing temperatures and time (*μ*g/mL).

Processing time (min)	120°C	140°C	160°C	180°C	200°C
1	154.67	147.12	94.92	92.72	92.41
3	154.05	112.96	8.26	0	0
5	152.48	105.68	0	0	0
10	144.29	104.13	0	0	0
15	138.34	78.04	0	0	0
20	137.19	74.35	0	0	0
25	134.58	67.58	0	0	0
30	134.39	19.36	0	0	0
35	134.20	0	0	0	0
40	131.51	0	0	0	0

**Table 3:** ^1^H (600 MHz) and^13^C (150 MHz) NMR data of compound **1**.

Position	**1**					Aconitine
	*δ*_H_ (*J* in Hz)	*δ*_C_	HMBC	NOESY	^1^H–^1^H COSY	*δ*_C_
1	3.06 m	83.5 d	C-10, C-17, 1-OCH_3_	H-3, H-5, H-10, 1-OCH_3_	H-2*α*, *β*	83.4 d
2*α*	2.32 m	34.2 t	C-4, C-11	——	H-1, H-2*β*, H-3	34.1 t
2*β*	2.18 m		C-4, C-11	——	H-1, H-2*α*, H-3	
3	3.68 m	71.5 d	C-1, C-18, C-19	H-1, H-5	H-2*α*, *β*	70.4 d
4	——	43.5 s	——	——	——	43.2 s
5	2.03 d (6.6)	48.1 d	C-3, C-7, C-10, C-17, C-18, C-19	H-1, H-3, H-18*α*, *β*	H-6	46.6 d
6	3.92 d (6.6)	84.1 d	C-4, C-8, C-17, 6-OCH_3_	H-8, 6-OCH_3_	H-5, H-7	82.3 d
7	2.73 m	42.5 d	C-5, C-9, C-11, C-15	6-OCH_3_	H-6, H-8	44.8 d
8	2.75 m	48.5 d	C-10, C-15, C-17	H-6, H-2′, 6′	H-7, H-9	92.0 s
9	2.78 m	38.6 d	C-12, C-13, C-15	——	H-8, H-10, H-14	44.2 d
10	2.15 m	43.1 d	C-5, C-8, C-17	H-1, H-14	H-9, H-12*α*, *β*	40.8 d
11	——	50.9 s	——	——	——	49.8 s
12*α*	2.71 m	36.1 t	C-9, C-11, C-13, C-14, C-16	——	H-10, H-12*β*	36.2 t
12*β*	2.26 m		C-9, C-11, C-13, C-16	H-14	H-10, H-12*α*	
13	——	76.5 s	——	——	——	74.0 s
14	5.16 d (5.1)	79.6 d	C-8, C-13, C-16, ArC=O	H-10, H-12*β*	H-9	78.9 d
15	——	211.8 s	——	——	——	78.9 d
16	3.29 s	89.2 d	C-8, C-12, C-14, C-15, 16-OCH_3_	H-17, 16-OCH_3_	——	90.1 d
17	2.81 s	61.9 d	C-5, C-6, C-8, C-10, C-19	H-16, H-21	——	61.0 d
18*α*	3.68 d (9.18)	76.2 t	C-3, C-5, C-19, 18-OCH_3_	H-5, H-19*α*, *β*, 18-OCH_3_	H-18*β*	77.1 t
18*β*	3.75 d (9.18)		C-3, C-5, C-19, 18-OCH_3_	H-5, H-19*α*, 18-OCH_3_	H-18*α*	
19*α*	2.37 d (10.98)	47.4 t	C-3, C-18, C-21	H-18*α*, *β*	H-19*β*	48.8 t
19*β*	2.89 d (10.98)		C-3, C-5, C-17	H-18*α*	H-19*α*	
21*α*	2.41 m	49.1 t	C-17, C-19	H-17	H-22	46.9 t
21*β*	2.44 m		C-17, C-19	H-17	H-22	
22	1.01 *t* (7.32)	13.3 q	——	——	H-21*α*, *β*	13.3 q
8-C=O	——	——	——	——	——	172.2 s
CH_3_	——	——	——	——	——	21.3 q
1-OCH_3_	3.23 s	56.1 q	C-1	H-1	——	55.6 q
6-OCH_3_	3.27 s	57.9 q	C-6	H-6, H-7	——	59.0 q
16-OCH_3_	3.61 s	61.7 q	C-16	H-16, H-2′, 6′	——	60.7 q
18-OCH_3_	3.28 s	59.2 q	C-18	H-18*α*, *ß*	——	57.9 q
ArC=O	——	167.1 s	——	——	——	165.9 s
1′	——	129.6 s	——	——	——	129.8 s
2′, 6′	7.94 d (7.32)	129.8 d	C-4′, ArC=O	H-8, 16-OCH_3_	H-3′, 5′	129.6 d
3′, 5′	7.40 *t* (7.32)	128.4 d	C-1′, ArC=O	——	H-2′, 6′, H-4′	128.6 d
4′	7.53 *t* (7.32)	133.3 d	C-2′, 6′	H-3′, 5′	H-3′, 5′	133.2 d

**Table 4:** ^1^H (600 MHz) and^13^C (150 MHz) NMR data of compound **2**.

Position	**2**					Aconitine
	*δ*_H_ (*J* in Hz)	*δ*_C_	HMBC	NOESY	^1^H–^1^H COSY	*δ*_C_
1	3.05 m	83.6 d	C-10, C-17, 1-OCH_3_	H-3, H-10, 1-OCH_3_	H-2*α*, *β*	83.4 d
2*α*	2.36 m	34.1 t	C-4	——	H-1, H-2*β*, H-3	34.1 t
2*β*	2.17 m		C-4, C-11	——	H-1, H-2*α*, H-3	
3	3.67 m	71.8 d	C-18, C-19	H-1, H-5	H-2*α*, *β*	70.4 d
4	——	43.7 s	——	——	——	43.2 s
5	2.04 d (6.6)	48.5 d	C-3, C-7, C-10, C-17, C-18, C-19	H-3, H-18*α*, *β*	H-6	46.6 d
6	3.89 d (6.6)	84.1 d	C-4, C-8, C-17, 6-OCH_3_	H-8, 6-OCH_3_	H-5, H-7	82.3 d
7	2.78 m	41.8 d	C-9, C-11, C-15	6-OCH_3_	H-6, H-8	44.8 d
8	2.65 m	49.4 d	C-10, C-15, C-17	H-6, H-16	H-7, H-9	92.0 s
9	2.80 m	38.7 d	C-12, C-13, C-15	——	H-8, H-10, H-14	44.2 d
10	2.22 m	44.8 d	C-8, C-17	H-1, H-14	H-9, H-12*α*, *β*	40.8 d
11	——	51.2 s	——	——	——	49.8 s
12*α*	3.02 m	32.9 t	C-11, C-13, C-14, C-16	——	H-10, H-12*β*	36.2 t
12*β*	1.83 m		C-11, C-13, C-16	H-14	H-10, H-12*α*	
13	——	77.5 s	——	——	——	74.0 s
14	5.41 d (5.16)	78.5 d	C-8, C-13, C-16, ArC=O	H-10, H-12*β*	H-9	78.9 d
15	——	211.7 s	——	——	——	78.9 d
16	3.85 s	86.1 d	C-12, C-15, 16-OCH_3_	H-8, H-2′, 6′	——	90.1 d
17	2.98 s	61.6 d	C-1, C-6, C-10, C-19	H-21	——	61.0 d
18*α*	3.69 d (9.18)	76.8 t	C-3, C-5, C-19, 18-OCH_3_	H-5, H-19*α*, *β*, 18-OCH_3_	H-18*β*	77.1 t
18*β*	3.75 d (9.18)		C-3, C-5, C-19, 18-OCH_3_	H-5, H-19*α*, 18-OCH_3_	H-18*α*	
19*α*	2.39 d (10.98)	47.4 t	C-3, C-5, C-18, C-21	H-18*α*, *β*	H-19*β*	48.8 t
19*β*	2.90 d (10.98)		C-3, C-5, C-17	H-18*α*	H-19*α*	
21*α*	2.45 m	49.0 t	C-17, C-19	H-17	H-22	46.9 t
21*β*	2.52 m		C-17, C-19	H-17	H-22	
22	1.04 *t* (7.38)	13.3 q	——	——	H-21*α*, *β*	13.3 q
8-C=O	——	——	——	——	——	172.2 s
CH_3_	——	——	——	——	——	21.3 q
1-OCH_3_	3.24 s	56.1 q	C-1	H-1	——	55.6 q
6-OCH_3_	3.24 s	57.8 q	C-6	H-6, H-7	——	59.0 q
16-OCH_3_	3.80 s	62.3 q	C-16	——	——	60.7 q
18-OCH_3_	3.29 s	59.2 q	C-18	H-18*α*, *β*	——	57.9 q
ArC=O	——	166.1 s	——	——	——	165.9 s
1′	——	129.4 s	——	——	——	129.8 s
2′, 6′	7.97 d (7.32)	129.7 d	C-4′, ArC=O	H-16	H-3′, 5′	129.6 d
3′, 5′	7.46 *t* (7.32)	128.6 d	C-1′, ArC=O	——	H-2′, 6′, H-4′	128.6 d
4′	7.60 *t* (7.32)	133.6 d	C-2′, 6′	H-3′, 5′	H-3′, 5′	133.2 d

**Table 5:** Comparison of cardiotoxicity between aconitine and its converted products (mean ± SD).

Compound	Dose (mg/kg)	VPB incubation period (s)	VT incidence (%)	Mortality (%)
Aconitine	0.03	97.7 ± 23.7	100	0
16-Epi-pyroaconitine	0.03	—	0	0
Pyroaconitine	0.03	—	0	0
	0.13	134.1 ± 36.5	80	10

“—” means that no arrhythmias occurred within 30 min after the injection of the experimental compounds.

**Table 6:** Chi-square analysis for the incidence of VT of 16-epi-pyroaconitine.

Dose (mg/kg)	VT (frequency)	Without VT (frequency)	*χ* ^2^	*P*
0.05	11	4	10.023	0.007
0.15–0.25	10	22		
0.30	3	11		

**Table 7:** Chi-square analysis of arrhythmia inhibition rate between groups.

Dose (mg/kg)	Arrhythmia (frequency)	Without arrhythmia (frequency)	*χ* ^2^	*P*
0.05	15	2	8.442	0.038
0.15	18	14		
0.25	14	13		
0.30	14	4

## Data Availability

The data used to support the article are available within the article or Supplementary Materials.

## Supplementary Materials

Supplementary Materials
*Figures S1–S27*. ^1^H-NMR, ^13^C NMR, HSQC, ^1^H–^1^H COSY, HMBC, NOESY, and HR-ESI-MS original spectra of compounds **1** and **2**. *Figure S28*. Purity test results for compounds **1** and **2**.
