# Supplementary material for: Attenuated Structural Transformation of Aconitine during Sand Frying Process and Antiarrhythmic Effect of Its Converted Products
Source: Evid Based Complement Alternat Med. 2021 Oct 25;2021:7243052. doi: 10.1155/2021/7243052 (PMC8560236; doi:10.1155/2021/7243052)
Supplement: Supplementary Materials — Figures S1–S27. 1H-NMR, 13C NMR, HSQC, 1H–1H COSY, HMBC, NOESY, and HR-ESI-MS original spectra of compounds 1 and 2. Figure S28. Purity test results for compounds 1 and 2. [file 7243052.f1.pdf]

Supplementary Materials for

Attenuated Structural Transformation of Aconitine During Sand Frying Process and Anti-arrhythmic Effect of Its Converted Products

Yu-Jie Wang,[1] Pei Tao,[2] and Yan Wang[2]

[1] School of Ethnic Medicine, Chengdu University of Traditional Chinese Medicine, Chengdu 611137, China.
[2] School of Pharmacy, Chengdu University of Traditional Chinese Medicine, Chengdu 611137, China.

Correspondence should be addressed to Yu-Jie Wang; superwangyj@126.com

This PDF file includes:

Figures S1 to S28

Content

1.1 Structure elucidation of pyroaconitine (compound 1) 3

^{1}H NMR of pyroaconitine 3

^{13}C NMR of pyroaconitine 5

Key HSQC correlations of pyroaconitine 6

Key HMBC correlations of pyroaconitine 8

Key ^{1}H-^{1}H COSY correlations of pyroaconitine 11

Key NOESY correlations of pyroaconitine 13

High-resolution ESI-MS of pyroaconitine 14

1.2 Structure elucidation of 16-epi-pyroaconitine (compound 2) 15

^{1}H NMR of 16-epi-pyroaconitine 15

^{13}C NMR of 16-epi-pyroaconitine 18

Key HSQC correlations of 16-epi-pyroaconitine 19

Key HMBC correlations of 16-epi-pyroaconitine 22

Key ^{1}H-^{1}H COSY correlations of 16-epi-pyroaconitine 26

Key NOESY correlations of 16-epi-pyroaconitine 27

High-resolution ESI-MS of 16-epi-pyroaconitine 29

1.3 Purity test results of compounds 1 and 2 30

1.1 Structure elucidation of pyroaconitine (compound 1)

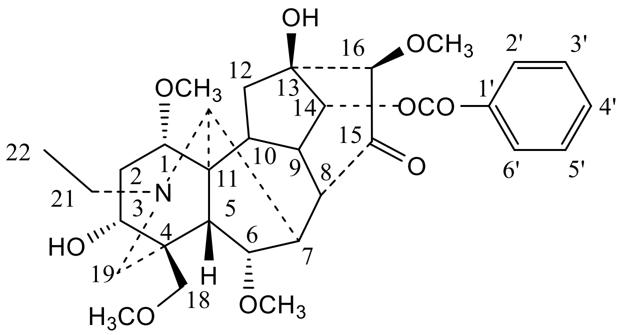

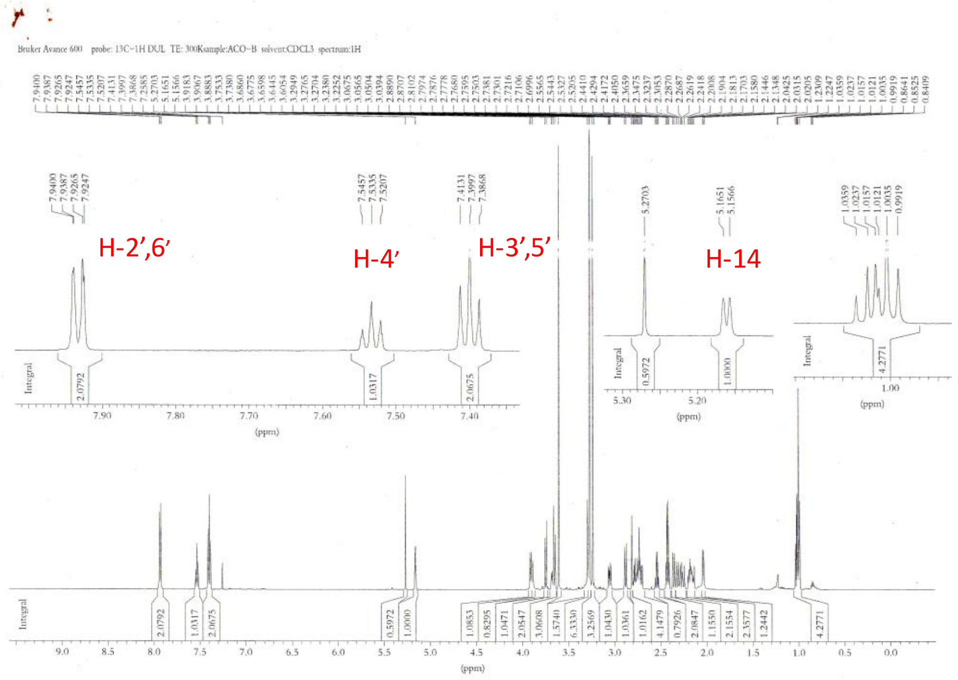

Figure S1. ^{1}H NMR of pyroaconitine

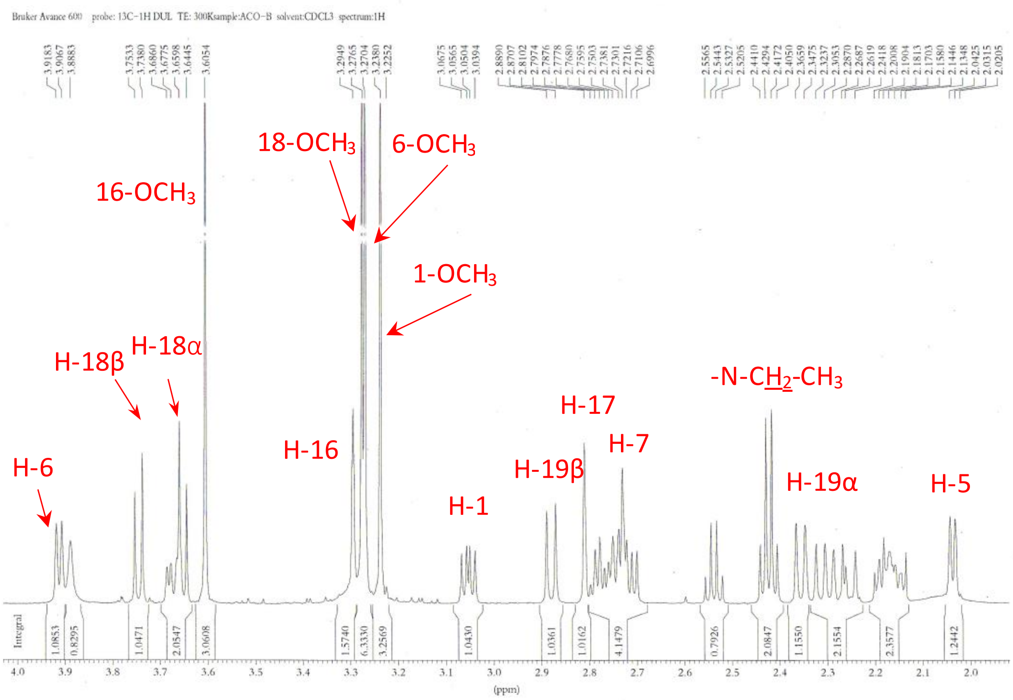

Figure S2. ^{1}H NMR of pyroaconitine (Detail)

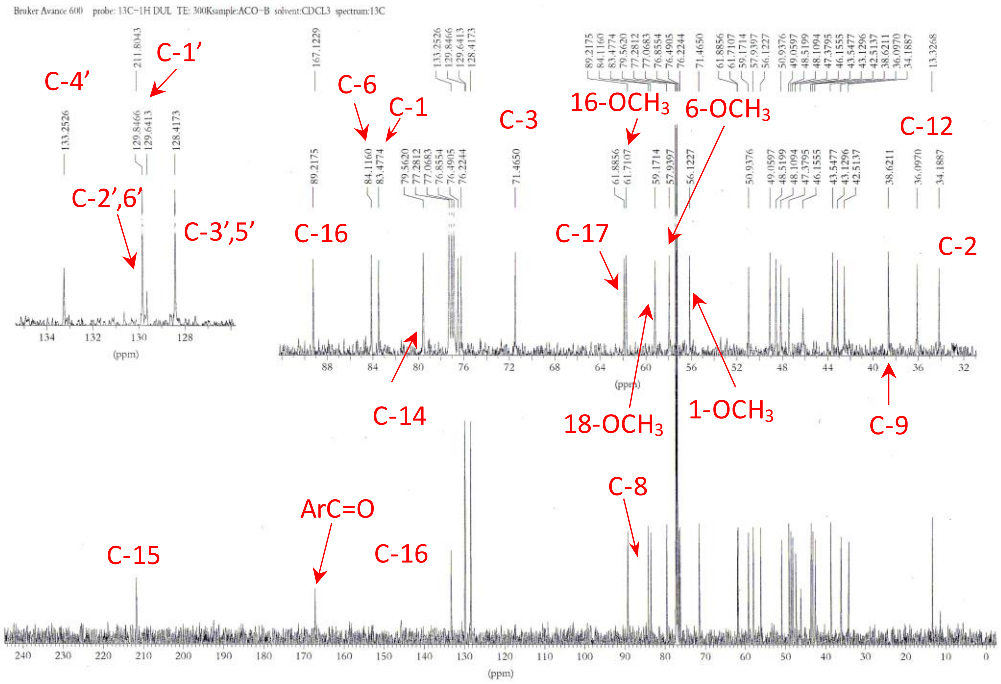

Figure S3. ^{13}C NMR of pyroaconitine

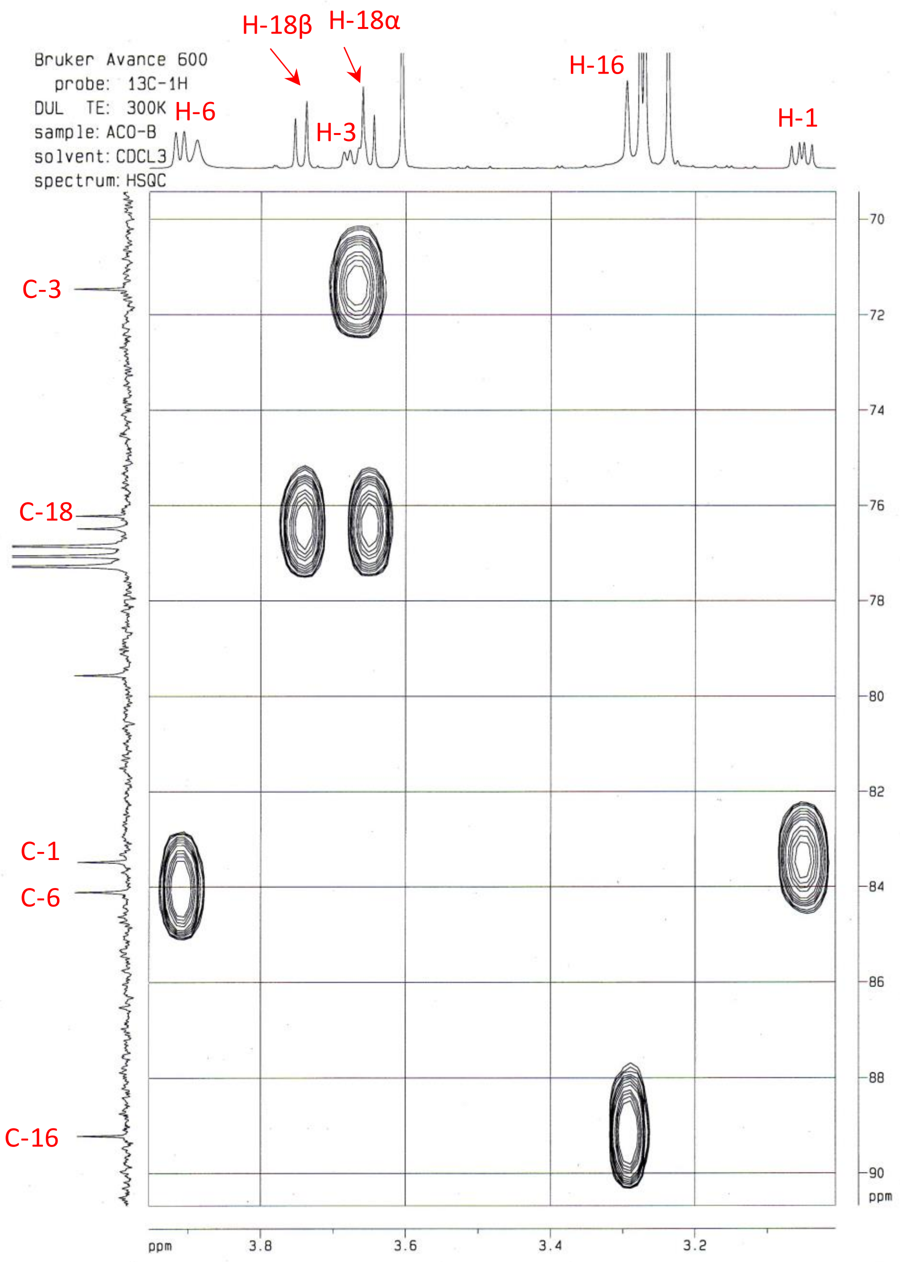

Figure S4. Key HSQC correlations of pyroaconitine (Detail 1)

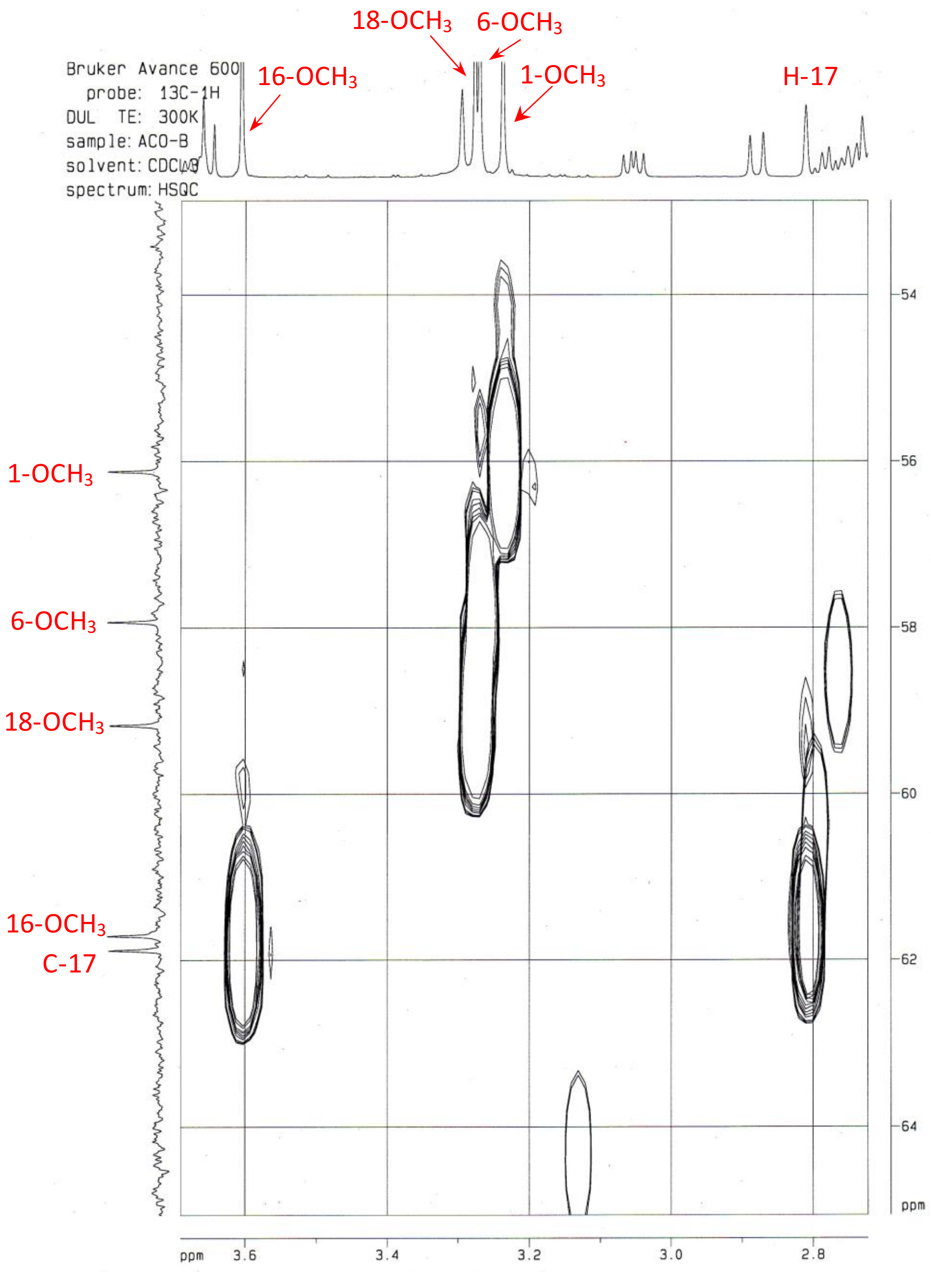

Figure S5. Key HSQC correlations of pyroaconitine (Detail 2)

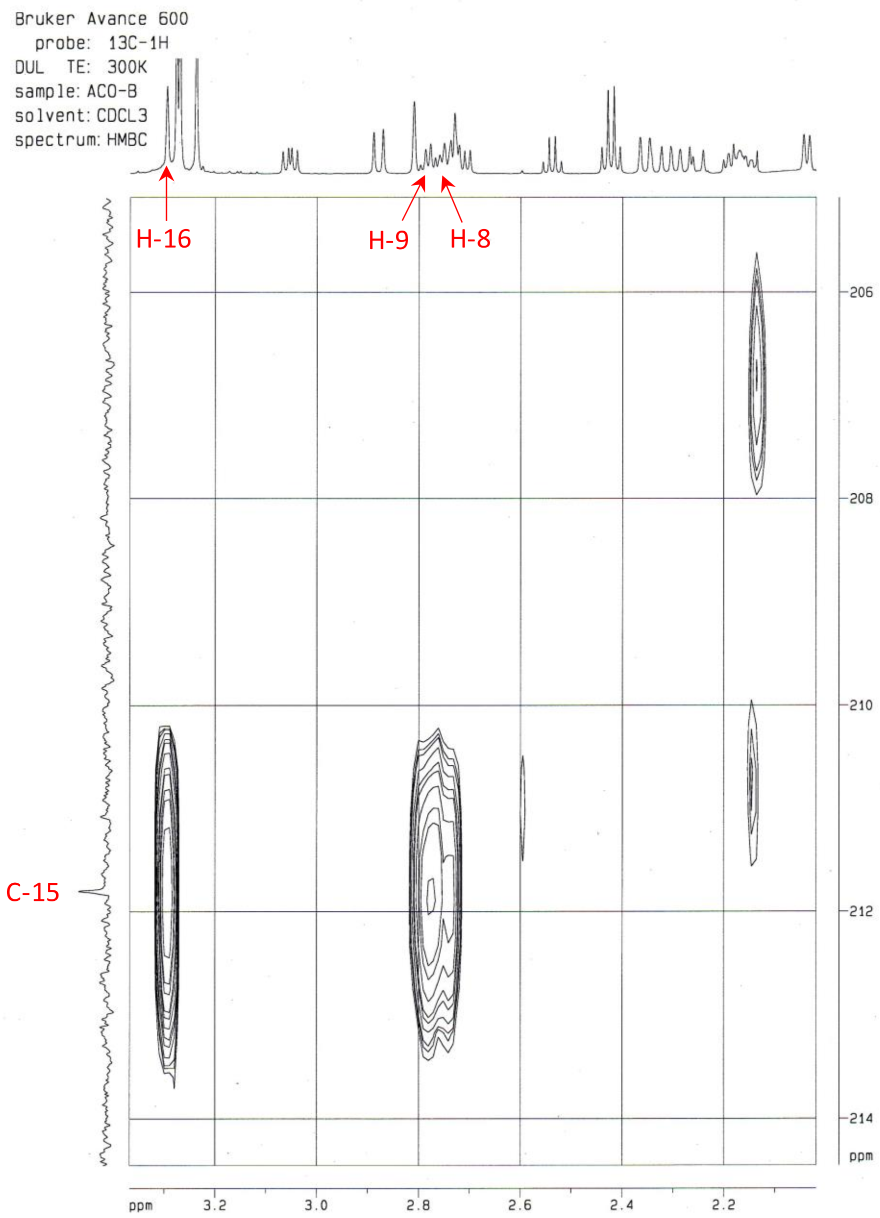

Figure S6. Key HMBC correlations of pyroaconitine (Detail 1)

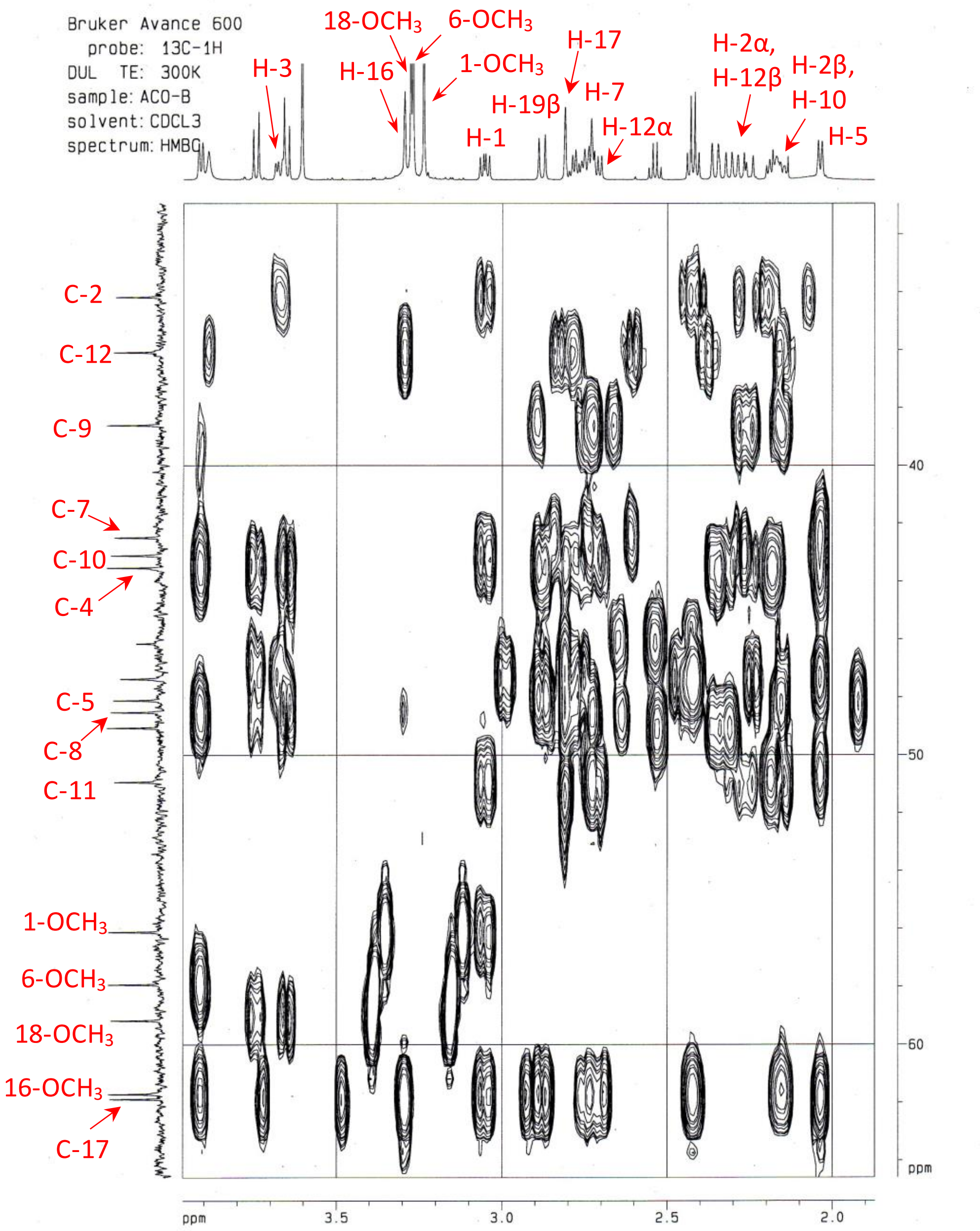

Figure S7. Key HMBC correlations of pyroaconitine (Detail 2)

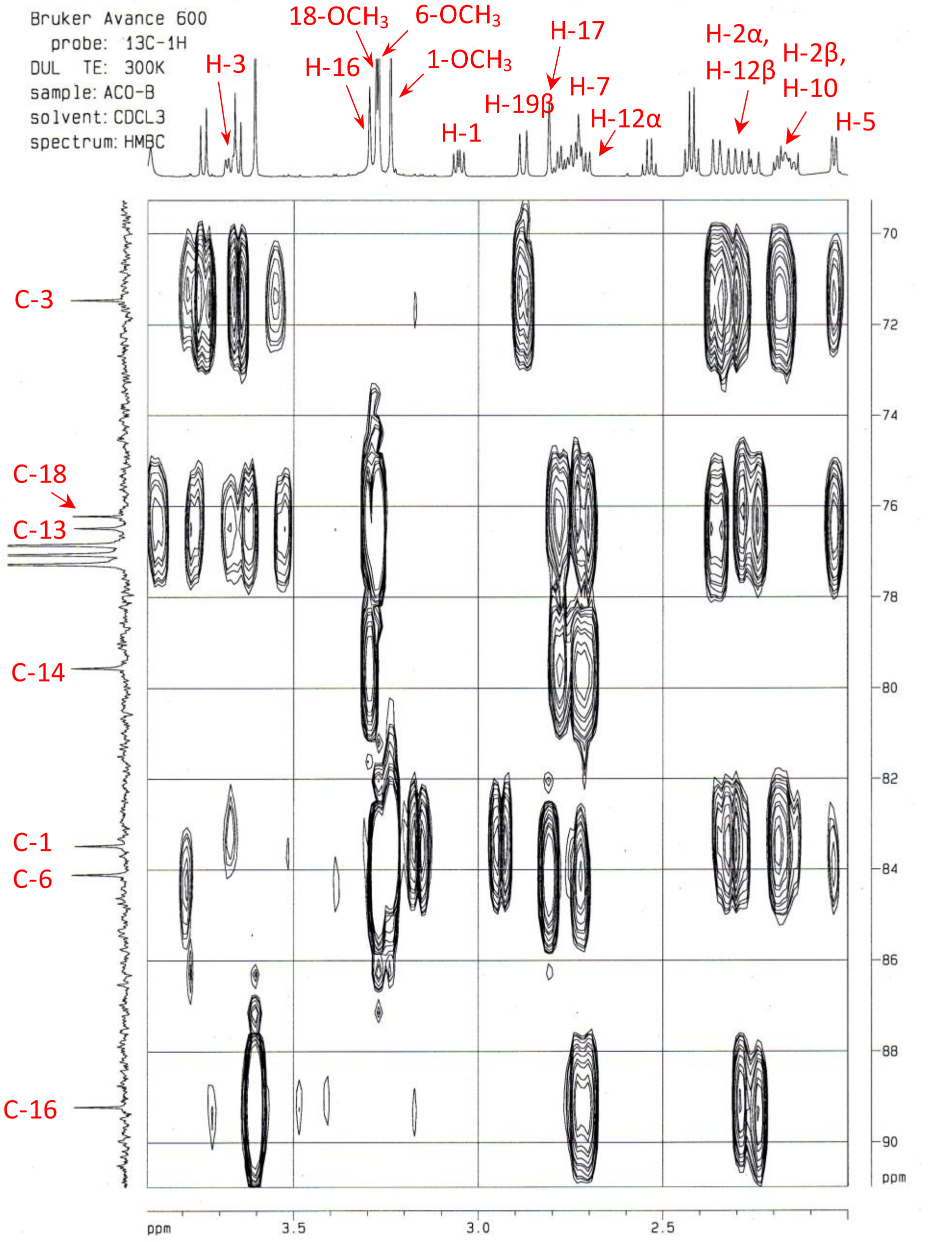

Figure S8. Key HMBC correlations of pyroaconitine (Detail 3)

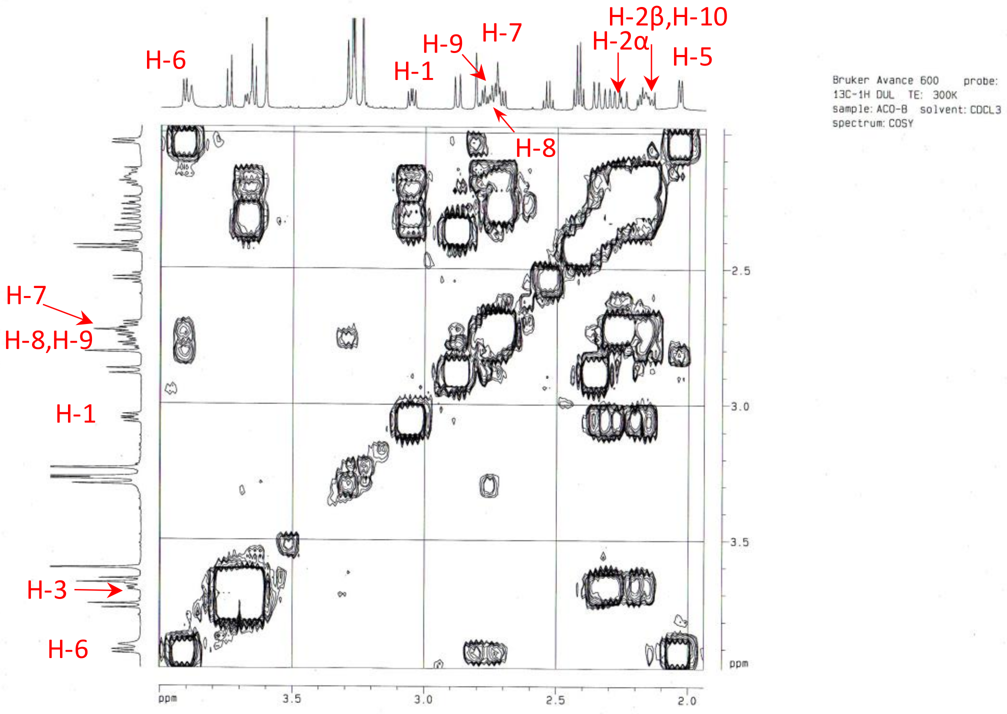

Figure S9. Key ^{1}H-^{1}H COSY correlations of pyroaconitine (Detail 1)

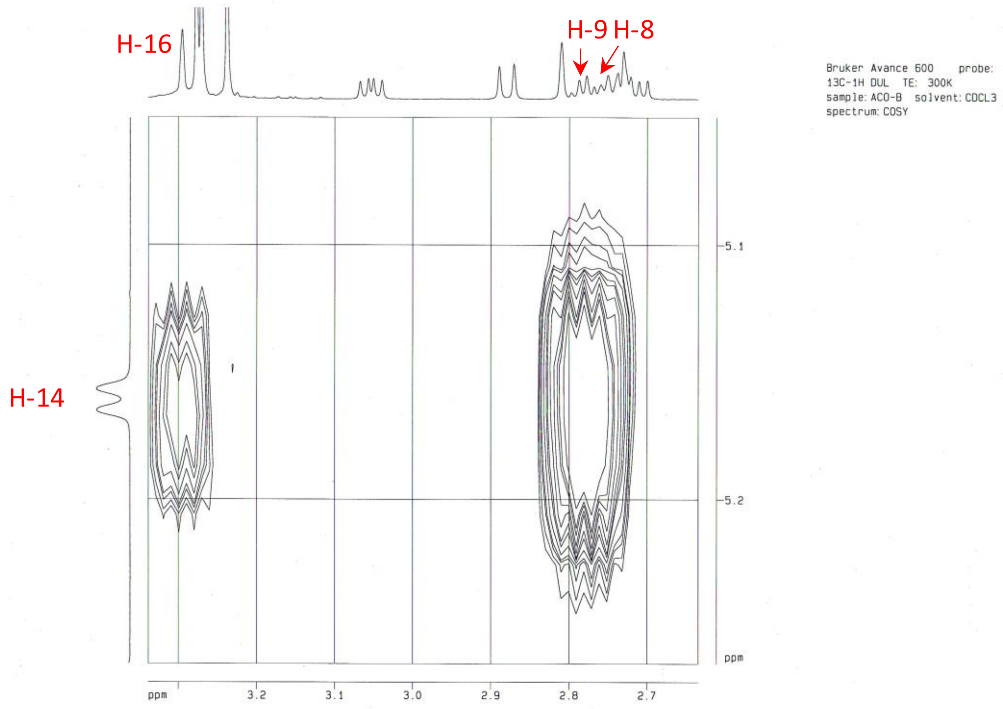

Figure S10. Key ^{1}H-^{1}H COSY correlations of pyroaconitine (Detail 2)

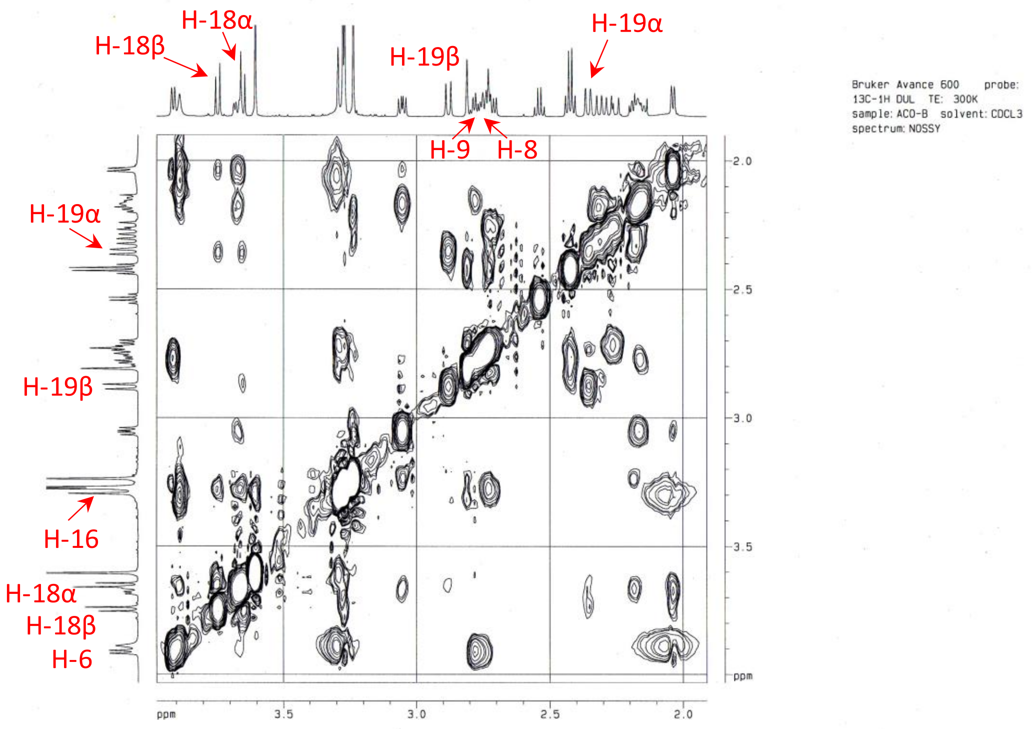

Figure S11. Key NOESY correlations of pyroaconitine (Detail 1)

Mass Spectrum SmartFormula Report

Analysis Info

Method	tune_low_NEW.m	Operator	Ma
Sample Name	ACO-B	Instrument / Ser#	micrOTOF-Q II 10203
Comment			

Acquisition Parameter

Source Type	ESI	Ion Polarity	Positive	Set Nebulizer	0.8 Bar
Focus	Not active	Set Capillary	4500 V	Set Dry Heater	180 °C
Scan Begin	50 m/z	Set End Plate Offset	-500 V	Set Dry Gas	6.0 l/min
Scan End	1500 m/z	Set Collision Cell RF	150.0 Vpp	Set Divert Valve	Source

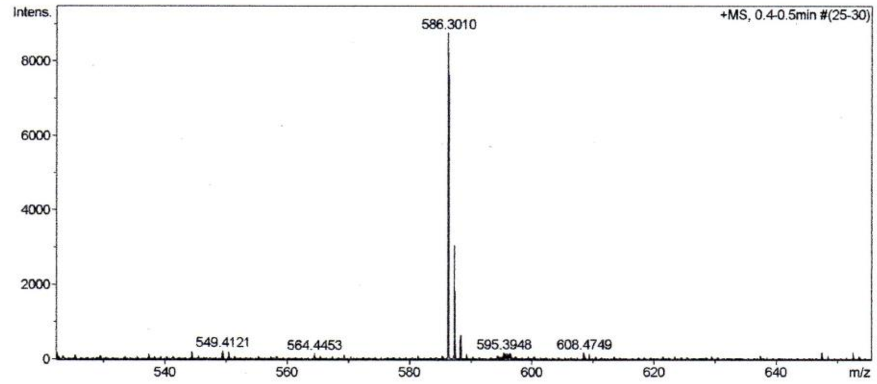

Meas. m/z	Formula	m/z	|err| [ppm]	mSigma	N-Rule	e⁻ Conf
586.3010						
	C 32 H 44 N O 9	586.3011	0.2	4.92	ok	even

Bruker Compass DataAnalysis 4.0 Page 1 of 1

Figure S12. High-resolution ESI-MS of pyroaconitine

1.2 Structure elucidation of 16-epi-pyroaconitine (compound 2)

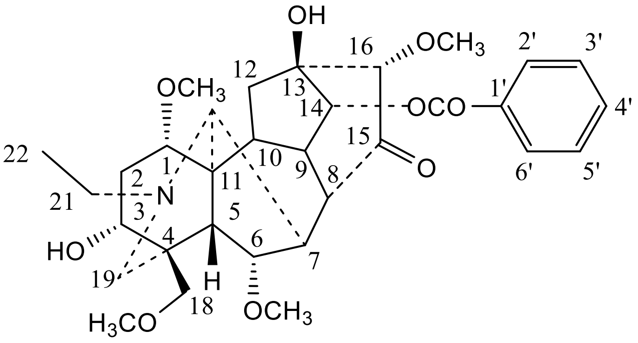

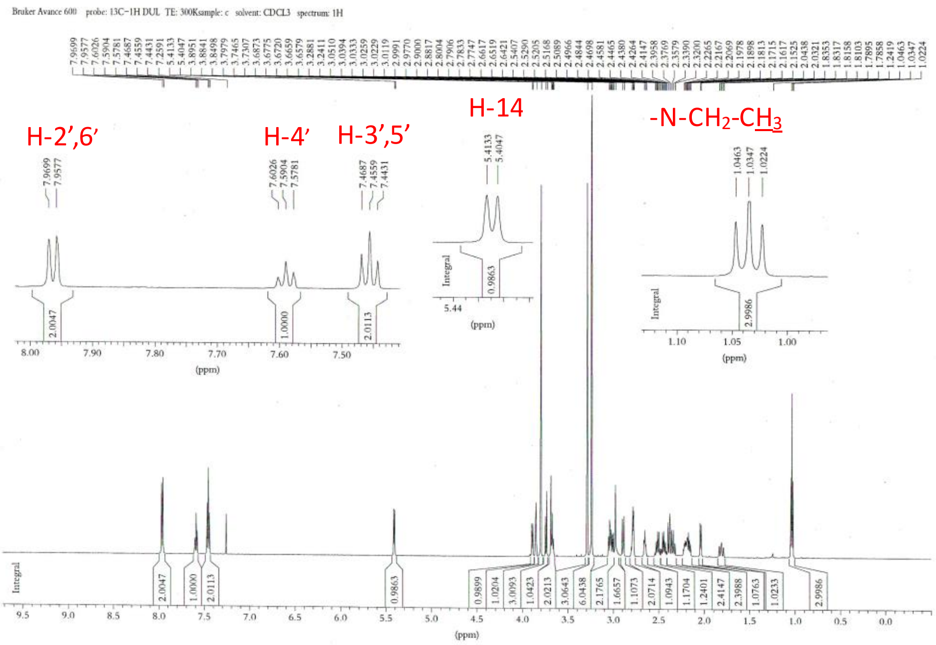

Figure S13. ^{1}H NMR of 16-epi-pyroaconitine

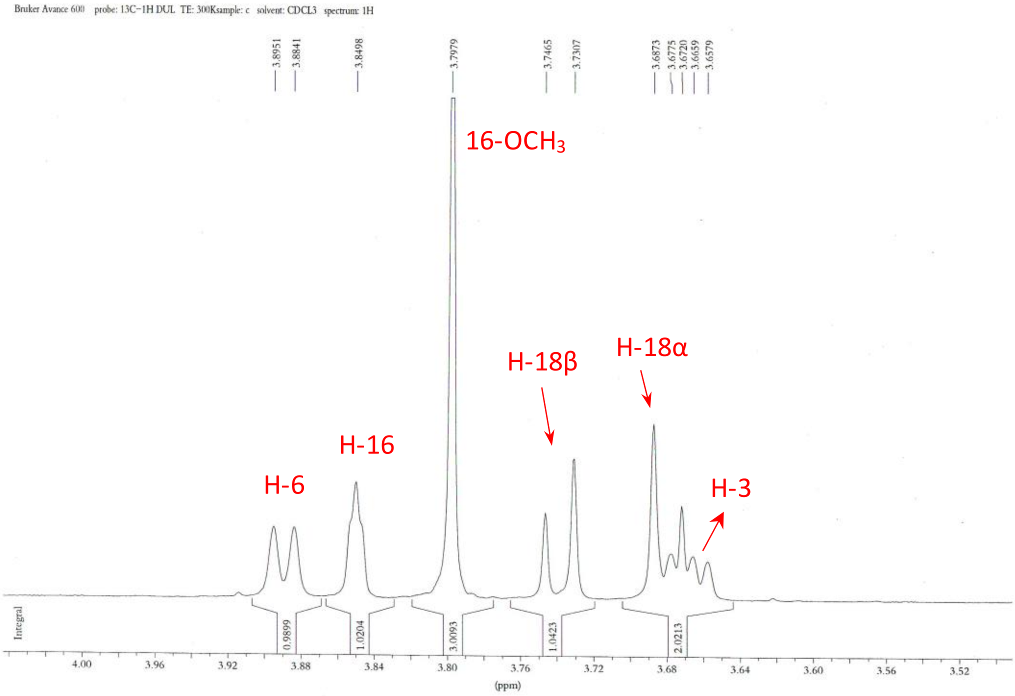

Figure S14. ^{1}H NMR of 16-epi-pyroaconitine (Detail 1)

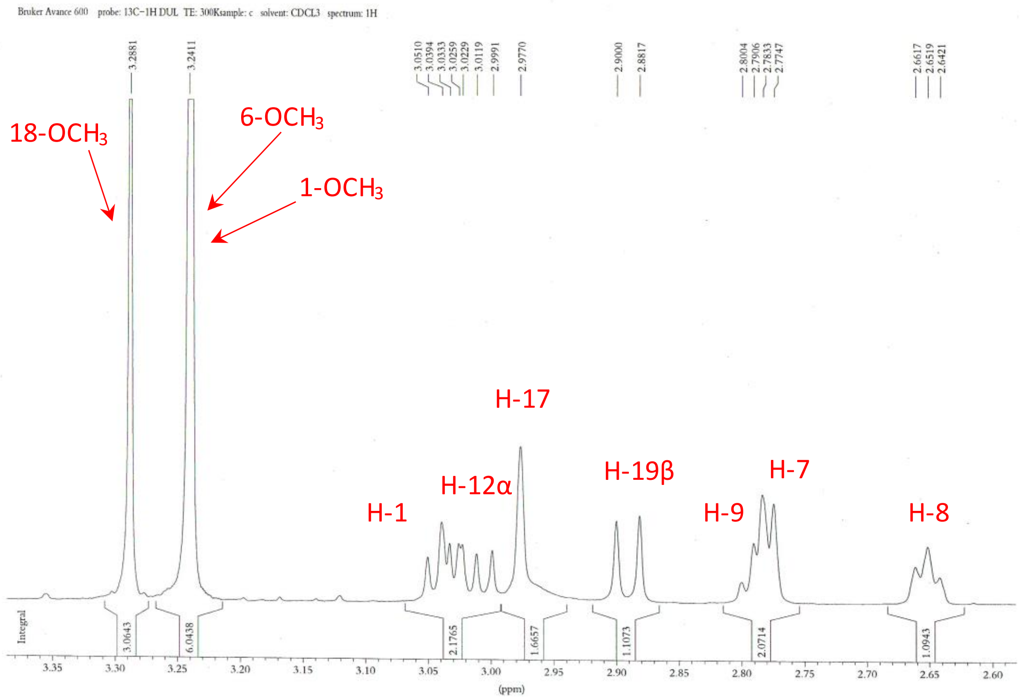

Figure S15. ^{1}H NMR of 16-epi-pyroaconitine (Detail 2)

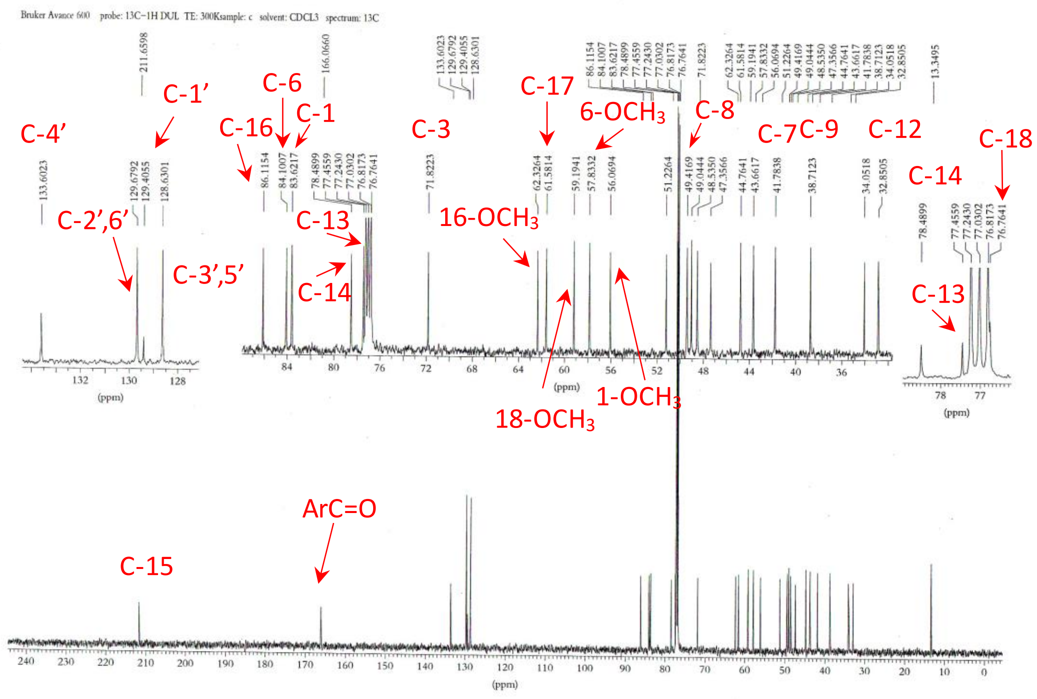

Figure S16. ^{13}C NMR of 16-epi-pyroaconitine

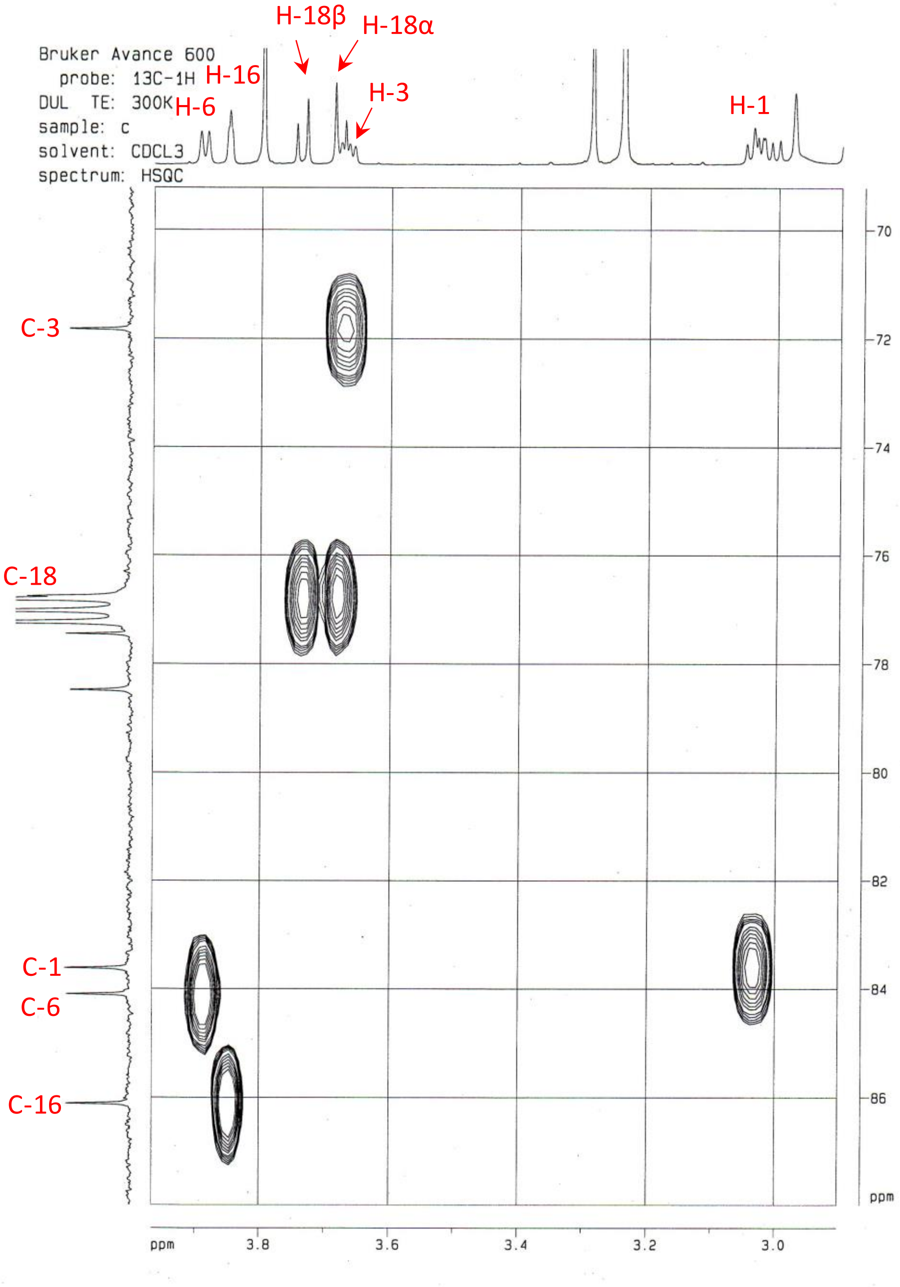

Figure S17. Key HSQC correlations of 16-epi-pyroaconitine (Detail 1)

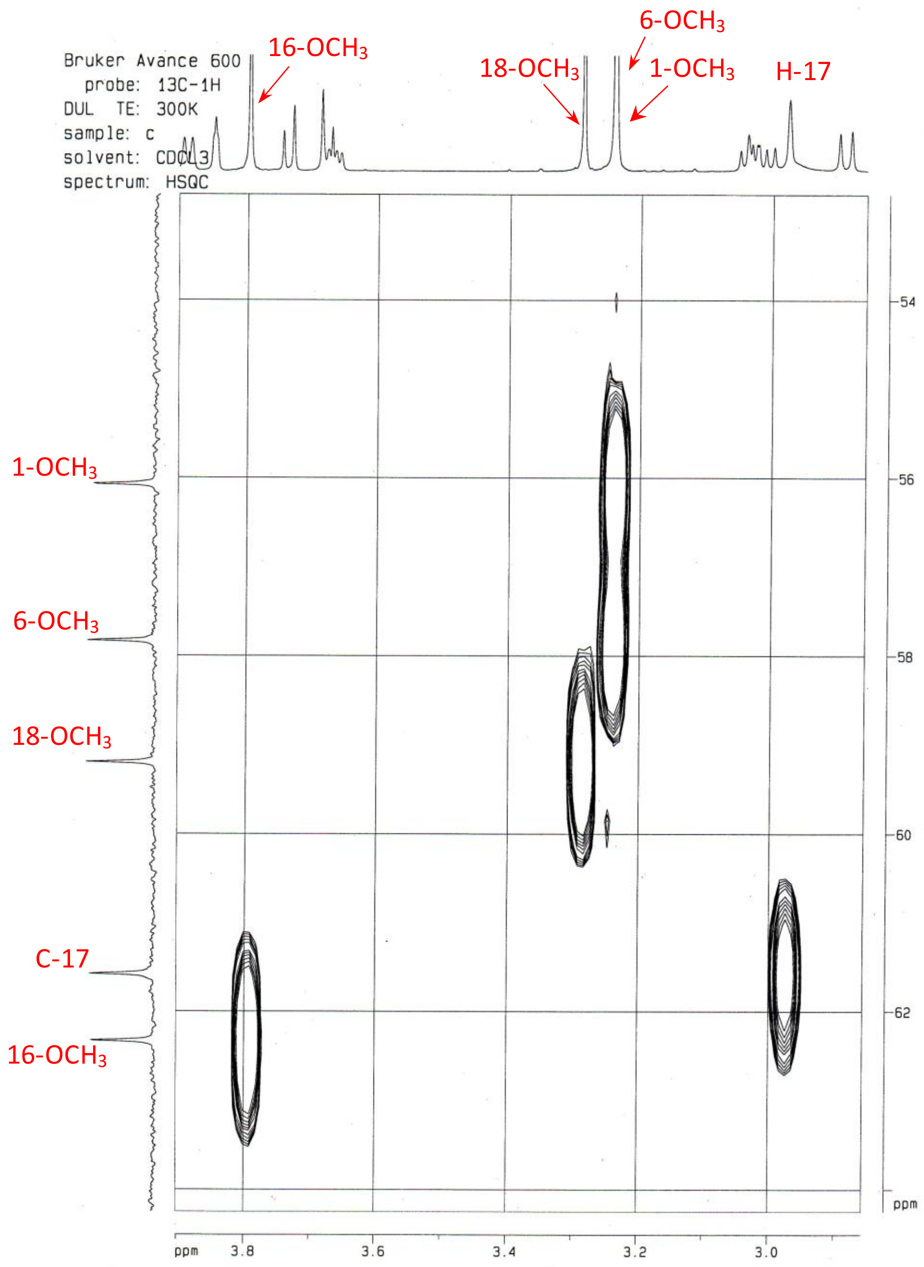

Figure S18. Key HSQC correlations of 16-epi-pyroaconitine (Detail 2)

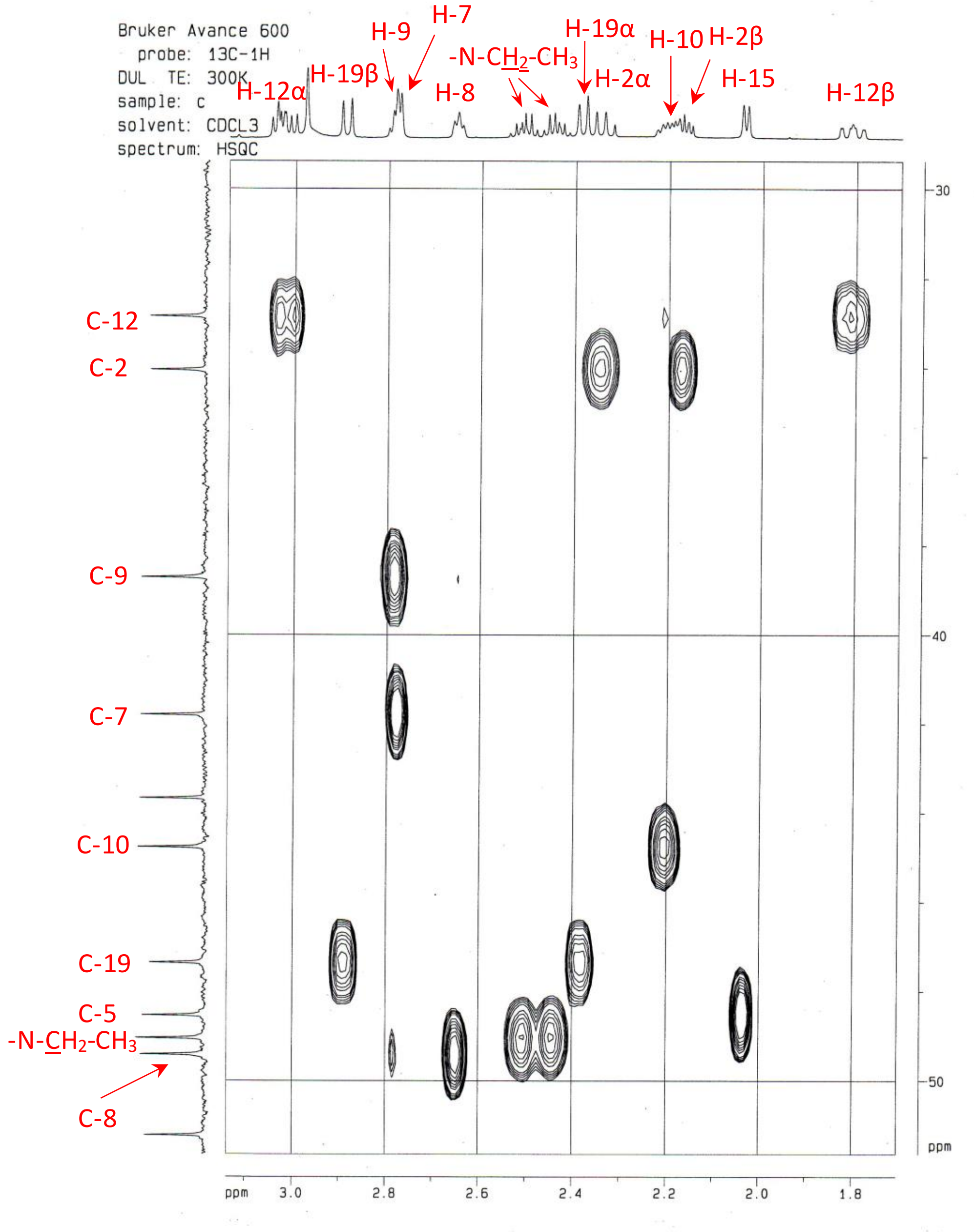

Figure S19. Key HSQC correlations of 16-epi-pyroaconitine (Detail 3)

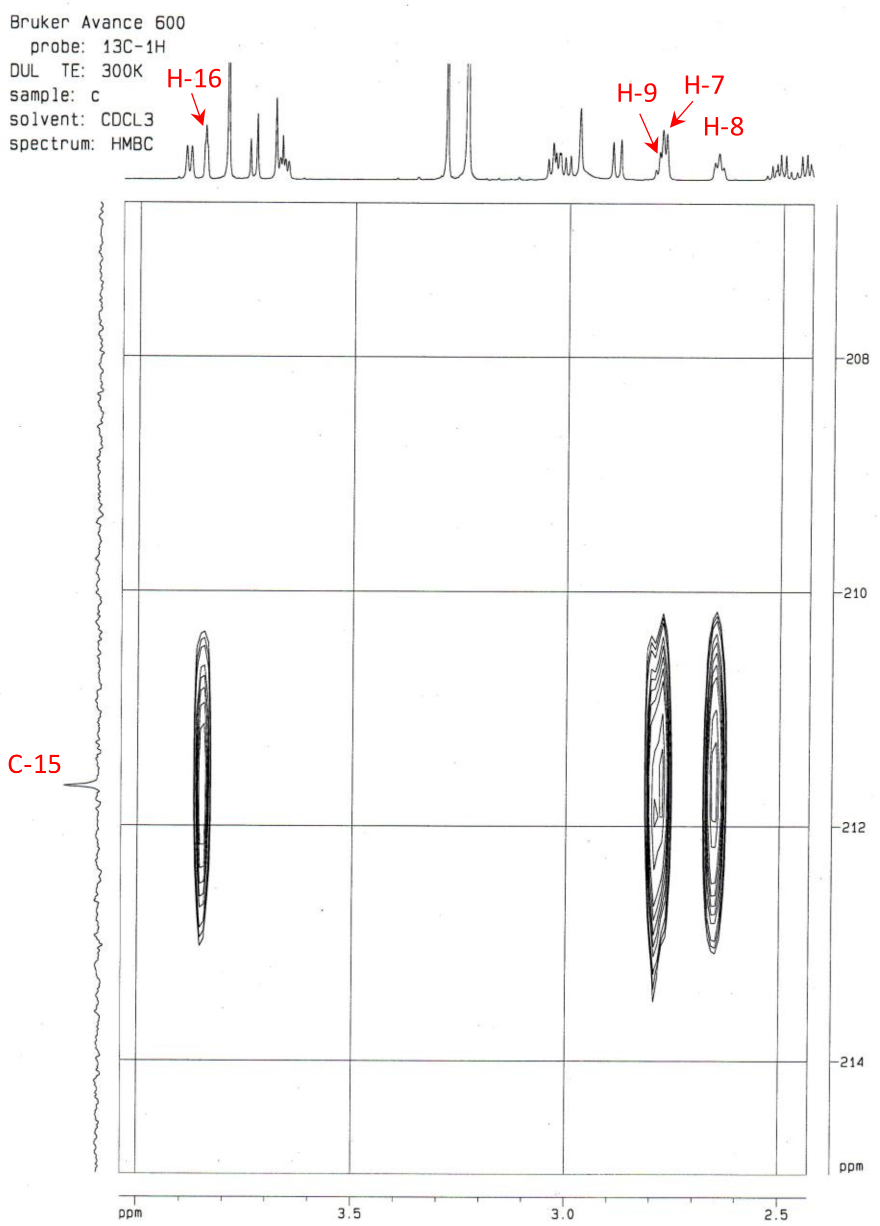

Figure S20. Key HMBC correlations of 16-epi-pyroaconitine (Detail 1)

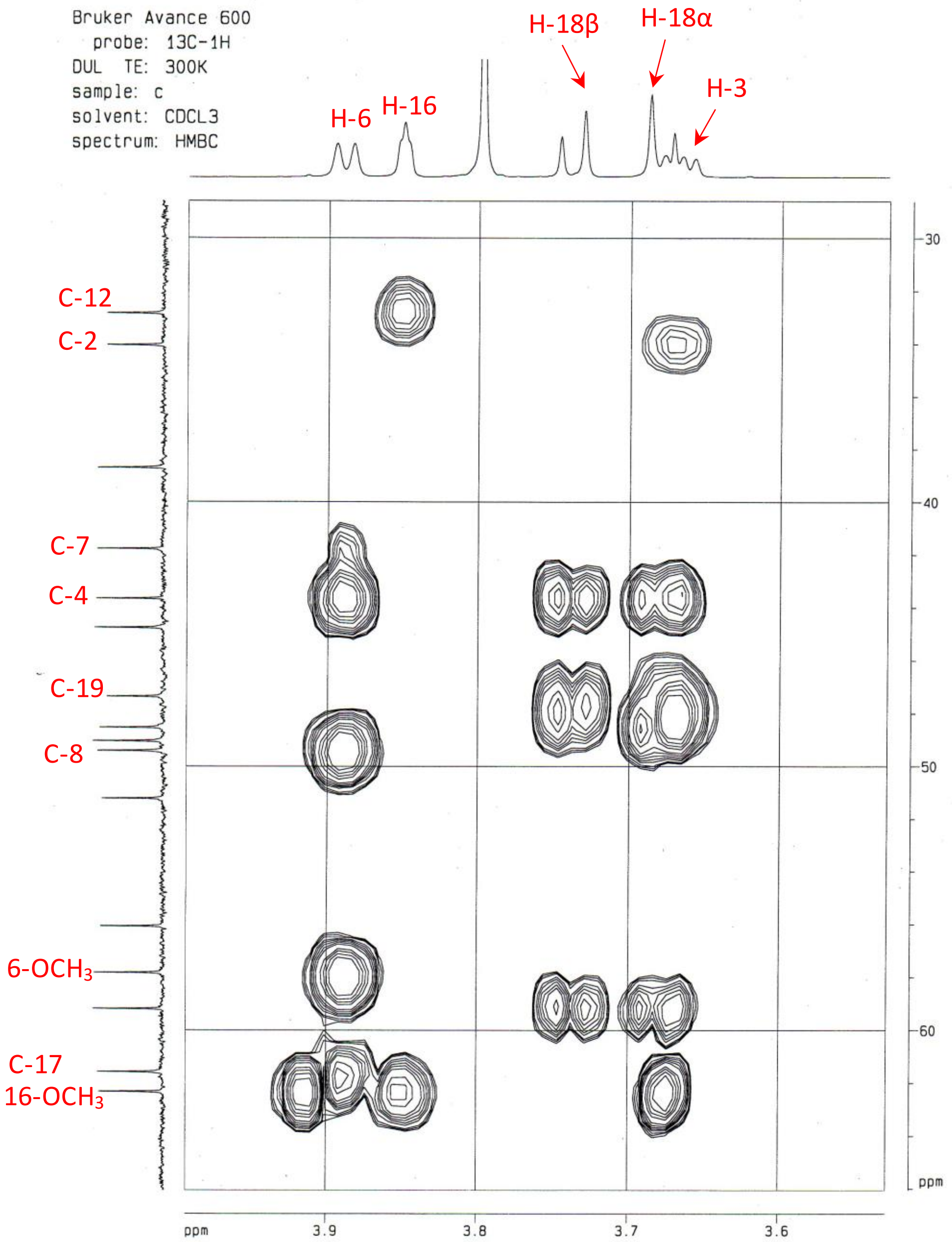

Figure S21. Key HMBC correlations of 16-epi-pyroaconitine (Detail 2)

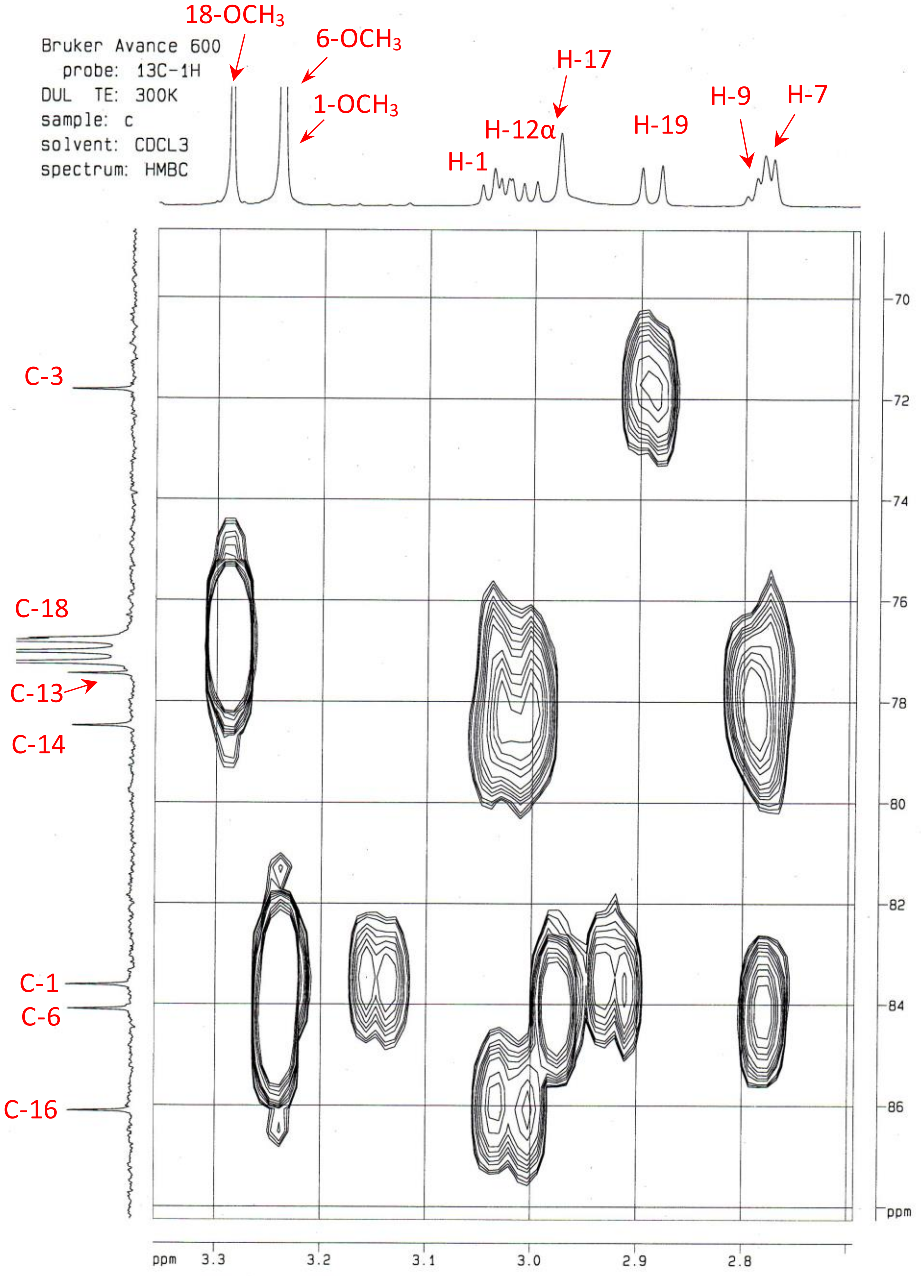

Figure S22. Key HMBC correlations of 16-epi-pyroaconitine (Detail 3)

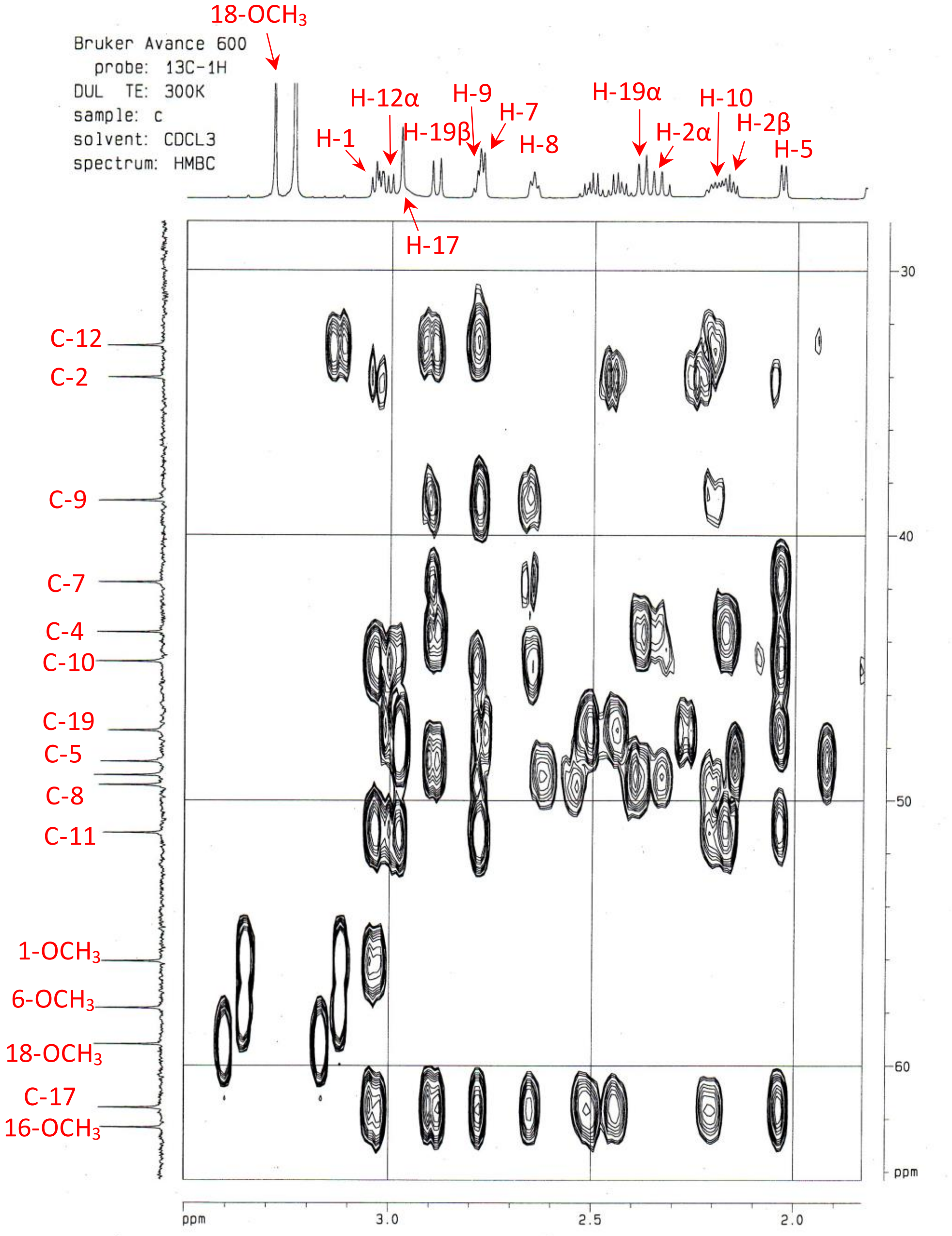

Figure S23. Key HMBC correlations of 16-epi-pyroaconitine (Detail 4)

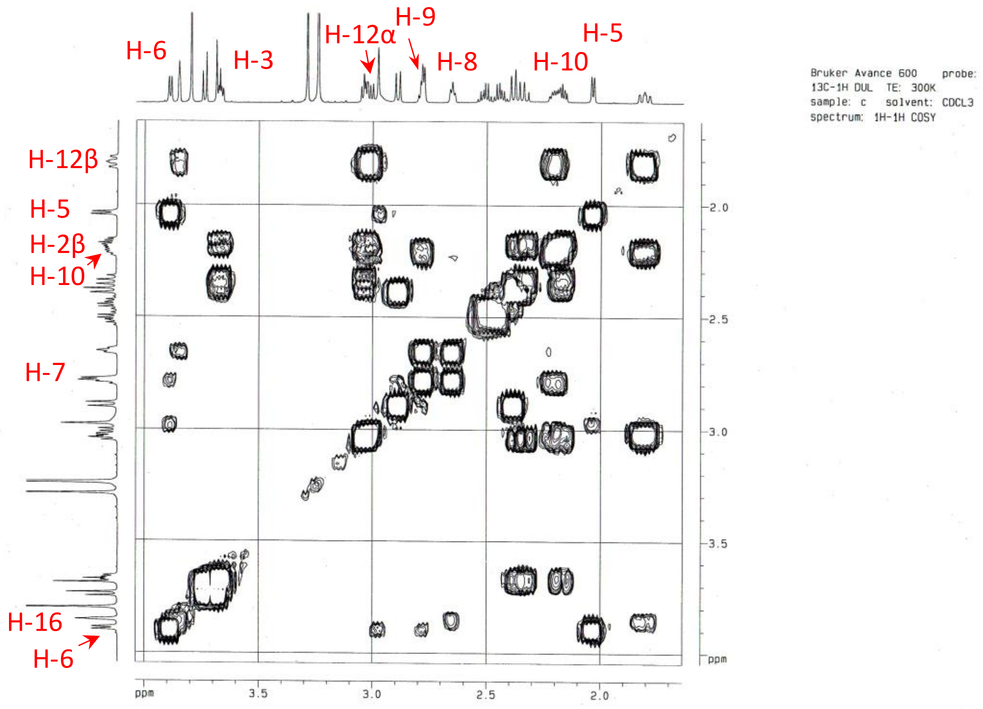

Figure S24. Key ^{1}H-^{1}H COSY correlations of 16-epi-pyroaconitine (Detail 1)

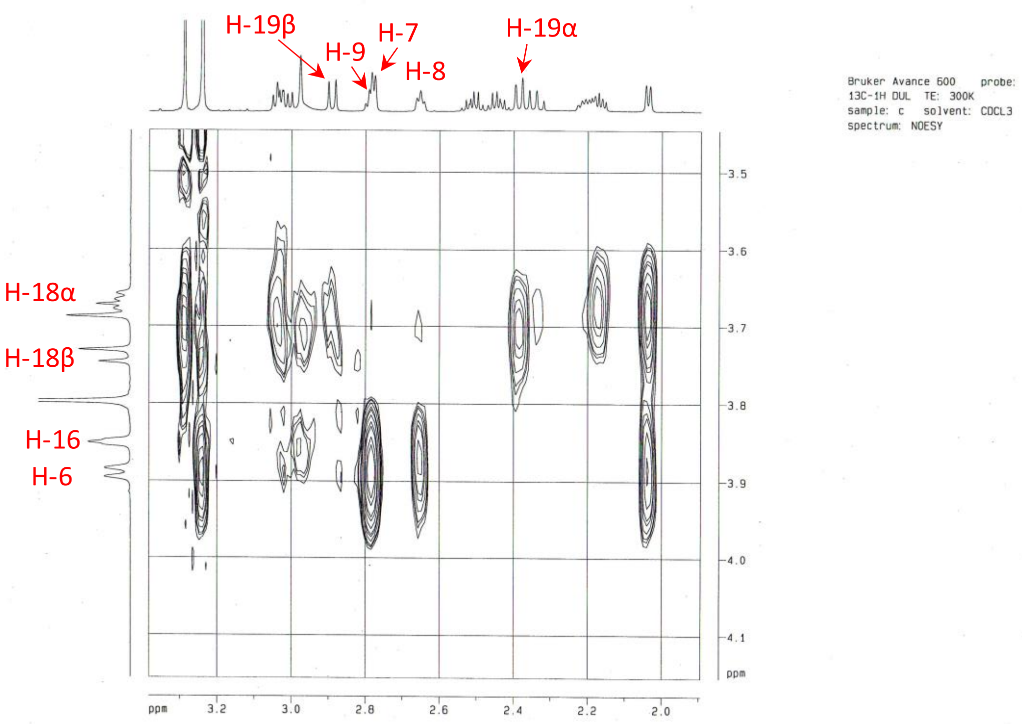

Figure S25. Key NOESY correlations of 16-epi-pyroaconitine (Detail 1)

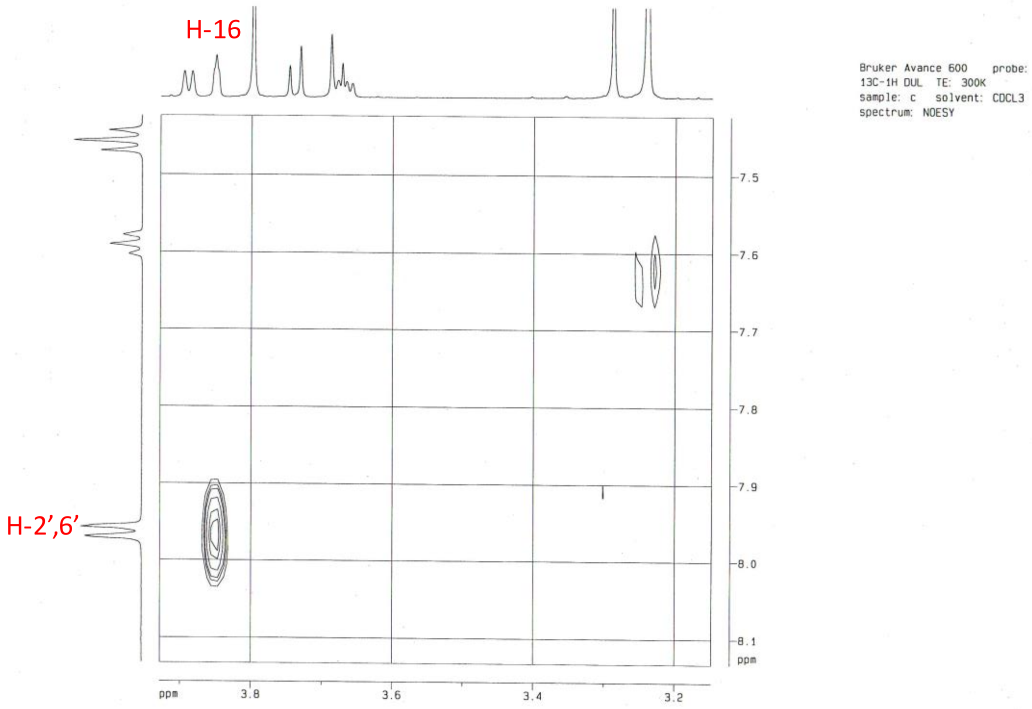

Figure S26. Key NOESY correlations of 16-epi-pyroaconitine (Detail 2)

Mass Spectrum SmartFormula Report

Analysis Info

Analysis Name	D:\Data\USER-2016\WYJ-C.d	Acquisition Date	5/31/2016 4:23:53 PM
Method	tune_low_NEW.m		
Sample Name	WYJ-C	Operator	Ma
Comment		Instrument / Ser#	micrOTOF-Q II 10203

Acquisition Parameter

Source Type	ESI	Ion Polarity	Positive	Set Nebulizer	0.3 Bar
Focus	Not active	Set Capillary	4500 V	Set Dry Heater	180 °C
Scan Begin	50 m/z	Set End Plate Offset	-500 V	Set Dry Gas	4.0 l/min
Scan End	1500 m/z	Set Collision Cell RF	150.0 Vpp	Set Divert Valve	Source

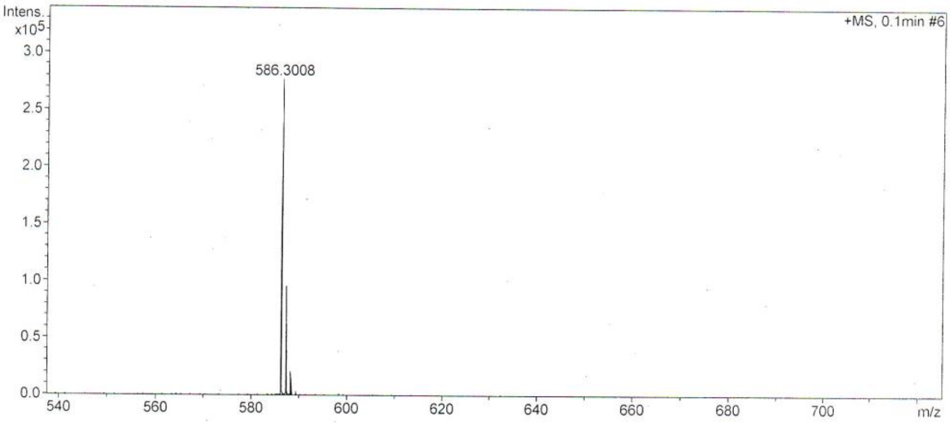

Meas. m/z	Formula	m/z	err [ppm]	mSigma	N-Rule	e⁻ Conf
586.3008						
	C 32 H 44 N O 9	586.3011	0.5	4.62	ok	even
	C 29 H 36 N 11 O 3	586.2997	-1.8	7.69	ok	even
	C 28 H 40 N 7 O 7	586.2984	-4.1	7.83	ok	even
	C 33 H 40 N 5 O 5	586.3024	2.8	17.00	ok	even

Bruker Compass DataAnalysis 4.0 Page 1 of 1

Figure S27. High-resolution ESI-MS of 16-epi-pyroaconitine.

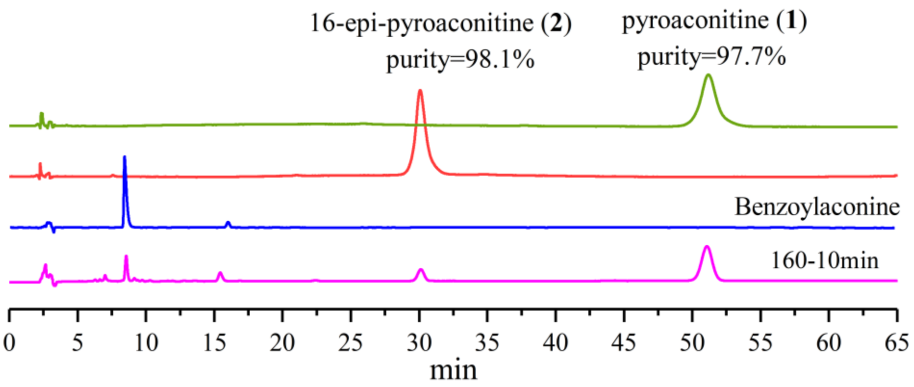

Figure S28. Purity test results of compounds **1** and **2**.
